# Therapeutic effects of medicinal and food-based traditional herbal couples on type 2 diabetes mellitus based on pharmacodynamics and pharmacokinetics

**DOI:** 10.3389/fphar.2025.1560271

**Published:** 2025-04-25

**Authors:** Yuhang Xu, Jing Li, Mengyao Cui, Xiaoliang Li, Hongyan Zhai, Deling Wu, Xiaoqin Chu

**Affiliations:** ^1^ School of Pharmacy, Anhui University of Chinese Medicine, Hefei, China; ^2^ Anhui Joyfar Pharmaceutical Research Institute Co., Ltd., Hefei, China; ^3^ Anhui Province Institute for Food and Drug Control, Hefei, China; ^4^ Bozhou University, Bozhou, China; ^5^ Anhui Provincial Key Laboratory of Traditional Chinese Medicine Decoction Pieces of New Manufacturing Technology, Hefei, China; ^6^ Institute of Pharmaceutics, Anhui Academy of Chinese Medicine, Hefei, China; ^7^ Anhui Province Key Laboratory of Pharmaceutical Preparation Technology and Application, Hefei, China; ^8^ Engineering Technology Research Center of Modern Pharmaceutical Preparation, Hefei, Anhui, China

**Keywords:** homologyofmedicineandfood, type 2 diabetes mellitus, cinnamomi ramulus, puerariae lobatae radix, gut flora, short-chain fatty acids, oral absorption

## Abstract

**Introduction:**

Cinnamomi Ramulus (CR) is the dried bark of Cinnamomum cassia Presl, Lauraceae. Puerariae Lobatae Radix (PLR) is the dried root of the Pueraria lobata (Wild.) Ohwi, Leguminosae. This Chinese herb couple come from the classic formula “Gui Zhi Ge Gen Tang,” which is included in the TCM classic “Treatise on Febrile Diseases.” Our previous studies have found that CR related herbal compound and PLR related herbal compound are useful in improving type 2 diabetes mellitus (T2DM), which is expected to be an antidiabetic candidate with fewer side effects. However the mechanism of action of CR-PLR on T2DM has not yet been fully elucidated.

**Methods:**

The decoction of CR-PLR was prepared by aqueous extraction method and the composition of it was analyzed using UPLC-Q-TOF-MS and HPLC. The T2DM model was established by intraperitoneal injection of streptozotocin, and the groups of drug administration were metformin, CR, PLR and CR-PLR groups, with continuous gastric gavage for 6 weeks, and the serological indexes were detected by ELISA. The abundance of rats’ gut flora was detected by 16s rDNA sequencing, and changes in the content of short-chain fatty acids (SCFAs) in feces of rats were detected by GC-MS; and the expression of G protein-coupled receptor43 (GPR43) and glucagon-like peptide-1 (GLP-1) proteins in colonic tissues of rats were detected by Western Blot. The pharmacokinetic behavior of CR-PLR was investigated in both normal and T2DM model rats. Caco-2/HT29 co-culture cell model was established in vitro, transepithelial electrical resistance (TEER) and ALP activity of epithelial cells were measured to evaluate cell model integrity and cell polarization, Alcian blue staining was used to verify the presence of mucus production, and CCK-8 was used to screen drug safe concentration. The bidirectional transport of puerarin was studied to investigate the transport mechanism of puerarin and the effect of leuric acid on puerarin transport.

**Results and discussion:**

The results indicated that CR-PLR can stimulate intestinal flora, increase the content of SCFAs, activate intestinal GPR43 protein, and promote the secretion of GLP-1 in intestinal L cells, which plays a therapeutic role in the treatment of T2DM. Additionally, cytology and pharmacokinetics experiments have proved that cinnamic acid (CA) can enhance the absorption and transport of puerarin (PUR) by inhibiting the efflux effects mediated by P-gp and MRP efflux transporters. The present study exhibites the scientific and reasonable menaning of this novel Chinese herb couple treating T2DM from the perspecives of pharmacodynamics and pharmacokinetics.

## Graphical Abstract

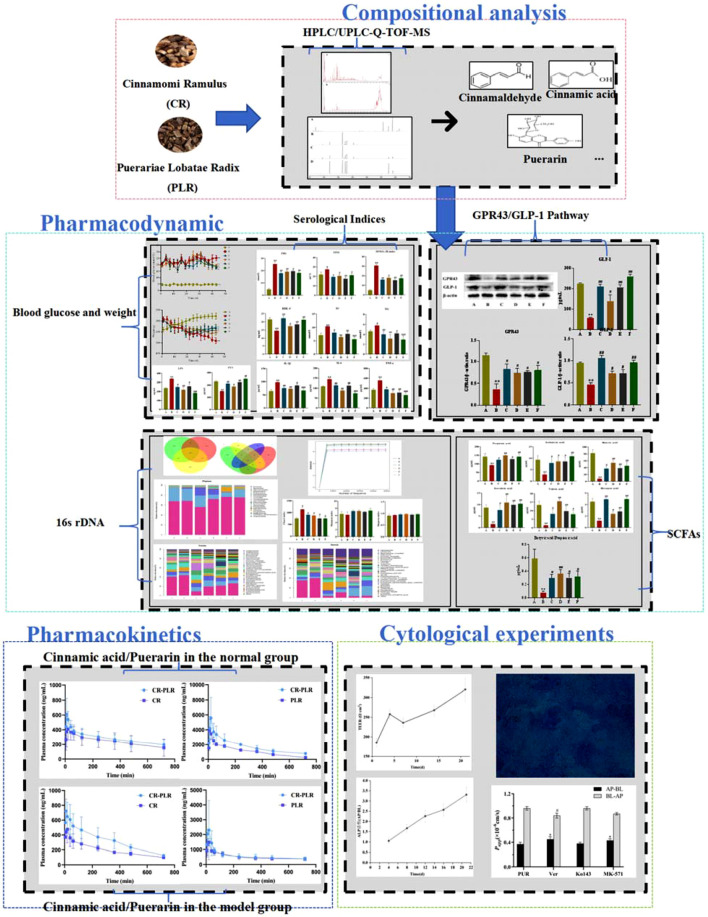

## 1 Introduction

Diabetes mellitus (DM) has become a public health concern, with Type 2 diabetes mellitus (T2DM) accounting for approximately 90% of diabetes (Zheng et al., 2018; Mizukami and Kudoh, 2022; Sun et al., 2023). The prevalence of T2DM has increased significantly in young adults and this rise is attributed to the increase in the number of obese people as more than 80% of people with T2DM are obese. T2DM is characterised by a decrease in insulin secretion by pancreatic β-cells (Huang et al., 2022) and the development of insulin resistance in the target tissues, including the liver, muscle and adipose tissues, which ultimately leads to hyperglycaemia (Liu et al., 2022). If left untreated, persistent hyperglycemia may lead to a range of complications, including cardiovascular disease, renal, retinal, and neurologic complications. Widespread drug therapy has been approved for the treatment of T2DM, with mechanisms mainly including reducing liver glucose production, stimulating insulin release, increasimg pancreatic function and sensitivity to insulin, delaying intestinal absorption of glucose, and regulation of insulin or glucagon secretion (Chen et al., 2023; Al-kuraishy et al., 2023; Al Jobori et al., 2017; Gilbert and Pratley, 2020; Lehmann and Hornby, 2016; Lim et al., 2023). However, the side effects of these drugs limit their therapeutic efficacy and clinical application in intolerant patients (Ansari et al., 2023).

Cinnamomi Ramulus (CR) is the dried bark of *Cinnamomum cassia Presl*, Lauraceae, while cinnamaldehyde is an allylaldehyde organic compound extracted from it, Cinnamaldehyde is easily metabolized to cinnamic acid (CA) in the body to produce medicinal effects. CR is not only of high medicinal value, but also as a spice. CR is processed into cinnamon powder, which is widely used in western countries for baking bread, snacks, etc. It is also used in large quantities for curing all kinds of meat foods such as ham. In China, the food industry with CR boiled, stewed, boiled all kinds of meat, to increase the aroma and flavor at the same time also used to process a variety of foods such as five-spice melon seeds, five-spice beans and so on. Cinnamon oil in the beverage and food industry, used as an additive and preservative, where “Coca-Cola,” “Pepsi” and other cola drinks can not be missing cinnamon oil (Deng, 2018). Some studies have shown that cinnamaldehyde has various pharmacological activities such as anti-inflammatory, antipyretic and analgesic, antitumour, antibacterial, hypoglycemic and anti-obesity (Pang et al., 2021; Zhao et al., 2015; Mendes et al., 2016; Doyle and Stephens, 2019). Interestingly, it was found that overexpression of protein tyrosine phosphatase-1B in tissue cells reduces the activity of protein tyrosine kinase, which prevents the insulin receptor from binding to insulin, which in turn causes insulin resistance (IR) and ultimately leads to T2DM, whereas cinnamaldehyde inhibits the activity of protein tyrosine phosphatase-1B, which can help to treat or prevent T2DM (Saifudin et al., 2013). Cinnamaldehyde also enhances antioxidant defence against reactive oxygen species generated under hyperglycemic conditions, protecting islet B cells from loss and producing hypoglycemic effects (Subash-Babu et al., 2014; Jawale et al., 2016). Puerariae Lobatae Radix (PLR) is the dried root of the *Pueraria lobata* (Wild.) Ohwi, Leguminosae. It is commonly used in the treatment of external fever, thirst and other conditions (Chen et al., 2018). Puerarin (PUR) is the main component of PLR, which is known as “Asian Ginseng” and “Plant Gold,” and has various functions such as medicinal, edible, green and ecological, etc. In March 1998, the Chinese Ministry of Health recognized PLR as a dual-use plant. As medicine, it can treat many diseases with good efficacy; as food, it is rich in nutrients and can make a variety of delicious food (Duan, 2021). Meanwhile, PUR is widely used in the treatment of cardiovascular disease, cerebrovascular disease, cancer, Parkinson’s disease, Alzheimer’s disease, DM and diabetic complications (Liu et al., 2023; Duan et al., 2021; Kampa et al., 2023; Li et al., 2023). PLR can significantly alleviate IR and has a good prospect for the treatment of T2DM (Zhou et al., 2014). CR-PLR come from the classic formula “Gui Zhi Ge Gen Tang,” which is included in the TCM classic “Treatise on Febrile Diseases” and is widely used to treat colds and fevers. Modern pharmacological studies have shown that CR-PLR can treat a variety of conditions such as neurogenic cervical spondylosis (Liu, 2024), peripheral facial paralysis (Zhang, 2023), and inflammatory lung injury caused by the influenza virus (Du et al., 2022). Interestingly, some scholars have formulated their own TCM formulas with CR and PLR as the main drugs. It was found that the TCM group could significantly lower blood glucose, reduce glycosylated serum protein in the blood, and improve blood rheology in diabetic rats, thus providing a therapeutic effect on diabetic rats (Cao et al., 2019).

The composition of the gut flora is influenced by internal and external factors (e.g., host genetics, age, chronic diseases, medications, environment, and diet) (Dubey et al., 2018; Halkjaer et al., 2024; Gao et al., 2024), and healthy populations have a more diverse composition of gut flora, while patients with T2DM have a less diverse gut flora, with more conditionally pathogenic bacteria and fewer beneficial flora. The study points out that, changes in gut flora are associated with altered glucose homeostasis and play an important role in the development and progression of obesity and T2DM. The gut flora of the host influences body weight, bile acid metabolism, pro-inflammatory activity, IR, gut hormone regulation (Xia et al., 2021). Current research on herbal medicines has found that there is an interactive relationship between the gut flora and traditional Chinese medicine (TCM). On the one hand, the gut flora can transform the active ingredients in TCM, and on the other hand, these active ingredients can reverse the imbalance of gut flora, which can lead to the improvement of diabetes symptoms after the imbalance of gut flora is restored (Xie et al., 2021; Wu et al., 2022; Zheng et al., 2021; Huang et al., 2023). Gut flora and its metabolites are transmitted through multiple pathways involving physiological processes in the body, including immunomodulation, metabolism, and even brain function (Wang et al., 2022). Short-chain fatty acids (SCFAs) produced by gut flora through fermentation and binding to G protein-coupled receptors (GPR41/GPR43) also stimulate the release of Peptide YY (PYY) and GLP-1, improving IR and favouring the improvement of T2DM symptoms.

Recent studies have shown that low-grade chronic inflammation is one of the central factors in the development and progression of type 2 diabetes mellitus (T2DM) (Hotamisligil, 2006). In patients with T2DM, it is often accompanied by elevated pro-inflammatory factors (e.g., TNF-α, IL-6, IL-1β), which are not only closely associated with the development of insulin resistance, but also promote pancreatic β-cell dysfunction. Specifically, TNF-α exacerbates insulin resistance by activating the NF-κB signaling pathway and inhibiting the action of insulin (Donath and Shoelson, 2011). Similarly, elevated IL-6 and IL-1β have been shown to reduce insulin effectiveness by interfering with insulin signaling pathways, further promoting metabolic disorders and elevated blood glucose. In addition, short-chain fatty acids (SCFAs), which are products of gut flora metabolism, play an important role in regulating inflammation and GLP-1 metabolism. SCFAs, particularly acetic acid, propionic acid, and butyric acid, inhibit inflammatory responses and promote GLP-1 secretion from intestinal L-cells by binding to the GPR43 receptor (Zhang et al., 2023). GLP-1 not only contributes to the regulation of insulin secretion and glucose metabolism, but also improves insulin resistance. Therefore, GPR43, as a receptor for SCFA, regulates inflammation and GLP-1 levels and may have an important regulatory role in the development and treatment of T2DM.

In summary, in this study, a T2DM rat model was established to investigate the ameliorative effects of CR-PLR on the intestinal flora, related inflammatory factors and GPR 43/GLP-1 pathway in T2DM rats. In addition, model and normal animals were established to carry out relevant pharmacokinetic studies in animals in two different states. In order to explore the absorption status of CA and PUR in normal and model animals, and to verify whether there is a synergistic effect of CR and PLR by cytological experiments.

## 2 Materials and methods

### 2.1 Materials

3′-hydroxypuerarin, puerarin, 3′-methoxygeraniol, puerarin apigenin、cinnamic acid, cinnamaldehyde (Shanghai Yuanye Biotechnology Co.); CR, PLR (The First Affiliated Hospital of Anhui University of Chinese Medicine) All herbs have been authenticated by Mr. Yu Nianjun, Professor of TCM at Anhui University of Traditional Chinese Medicine; methanoic acid, propionic acid, isobutyric acid, butyric acid, isovaleric acid, valeric acid, 2-Methylvaleric acid, hexanoic acid, mannitol (Shanghai Aladdin Biochemical Technology Co.); methanol, acetonitrile (Thermo Fisher, Germany); metformin (Shijiazhuang Ealing Pharmaceutical Co.); ELISA kits (Wuhan Genome Biotechnology Co.); β-actin antibody (Beijing Zhongsui Jinqiao Biotechnology Co.); FFAR2 Polyclonal antibody (Wuhan Sanying Biotechnology Co.); Goat anti-mouse IgG-HRP, Goat anti-rabbit IgG-HRP (Wuhan Genome Biotechnology Co.); Anti-GLP-1 antibody (Abcam, USA); SDS-PAGE Colour Gel Rapid Preparation Kit、ECL Chemiluminescent Substrate Kit, RIPA lysate (Beijing Lanjieke Technology Co.); Analytical balance (Sartorius, Germany); HPLC (Thermo Fisher, Germany); UPLC-Q-TOF-MS (Waters Corporation, USA); GC-MS (Agilent, USA); LC-MS (SCIEX Corporation, USA).

### 2.2 Animals

Sprague Dawley (SD) rats, male, body weight 180–220 g, provided by the Laboratory Animal Centre of Anhui Medical University, Animal Qualification Certificate No.: SCXK (Anhui) 2022-001.

### 2.3 Preparation of the CR-PLR decoction

Weigh an appropriate amount of CR and PLR of the same quality into a round bottom flask, add 10 times the amount of ultrapure water, soak for 30 min, heat and cool reflux for 30 min. Then, filter the decoction through a filter and pour it into a beaker. Add 8 times the amount of ultrapure water to the circular bottom flask mentioned above, heat and cool it again, and reflux for 60 min. Then, filter the decoction through the filter and pour it into the beaker mentioned above. Combine it with the first water extract to obtain the decoction of CR-PLR.

The preparation method of CR aqueous extract and PLR aqueous extract is the same as that of CR-PLR aqueous extract.

### 2.4 Compositional analysis of CR-PLR decoction

#### 2.4.1 UPLC-Q-TOF-MS

Liquid phase conditions: Waters ACQUITY UPLC BEH C_18_ column (2.1 mm × 150 mm, 1.7 μm) was used, with the mobile phase of 0.05% formic acid in water (phase A)-acetonitrile (phase B), and the gradient elution (0∼35 min, 3%∼41% B; 35∼45 min, 41%∼100% B; 45∼48 min, 100% B; 48∼49 min, 100%∼3% B; 49∼55 min, 3% B).

An ESI source was used for mass spectrometry analysis. The ESI source operation parameters were as follows: scanning range: 100∼1,500 m/z; capillary voltage: +19 V/−15 V; capillary temperature: 350°C; ion spray voltage: +4 kV/−3 kV; tube lens voltage: +25 V/−30 V; high-purity nitrogen was used as the sheath and auxiliary gases, sheath flow rate: 60 arb; auxiliary flow rate: 20 arb; multi-stage mass spectrometry. High-purity helium was used as the collision cracking gas, collision energy: 30%∼55%. Scanning was performed by multiple reaction monitoring.

#### 2.4.2 HPLC

The column was Phenomenex Luna C_18_ (250 mm × 4.6 mm, 5 μm); the mobile phase was acetonitrile (A)-0.1% phosphoric acid (B) with the gradient elution condition (0∼3 min, 5%∼11% acetonitrile; 3∼15 min, 11% acetonitrile; 15∼25 min, 11%∼15% acetonitrile; 25∼31 min, 15%∼20% acetonitrile; 31∼39 min, 20% acetonitrile; 39∼43 min, 20%∼25% acetonitrile; 43∼48 min, 25%∼30% acetonitrile; 48∼52 min, 30%∼35% acetonitrile; 52∼57 min, 35%∼40% acetonitrile; 57∼6 min, 40%∼50% acetonitrile; 61∼66 min, 50%∼5% acetonitrile; 66∼70 min, 5% acetonitrile); the flow rate was 1.0 mL/min; the column temperature was 30°C; injection volume was 20 μL; detection wavelength was 245 nm.

#### 2.4.3 GC-MS

The column was Agilent HP-FFAP 30 m × 0.32 mm × 0.25 μm. The carrier gas was helium with purity >99.999%. The inlet temperature was 230°C. The injection volume was 1 μL. The split ratio was 30:1. The flow rate was 1.3 mL/min. The programmed temperature increase: the GC oven program had an initial temperature of 90°C held for 1 min and then ramped to 120°C with a heating rate of 10°C/min, held for 0.5 min and then ramped to 150°C at 15°C/min, held for 0.5 min and then ramped to 180°C at 15°C/min, held for 1 min. The transfer line and MS ion source were maintained at 230°C, respectively. The ion source was an electron bombardment source (EI) with an electron energy of 70 eV. The mass spectrometry was performed by a full scanning mode. The scanning range was 30∼300 m/z.

### 2.5 Pharmacodynamics

#### 2.5.1 Establishment of rat model of T2DM

The T2DM rat model was developed by intraperitoneal injection of streptozotocin (STZ). 60 male SD rats were acclimatised and reared for 1 week, then blood glucose and body weight were measured, and the rats were randomly divided into 10 in the normal group and 50 in the modelling group according to blood glucose and body weight. The T2DM rat was fed with high-fat chow for 4 weeks, and at the end of the 4 weeks, the rats were fasted for 12 h without food or water, and were injected with a one-time intraperitoneal injection of 35 mg/kg of 1% STZ solution. The normal group was fed with normal chow, and was given free access to drinking water, and was injected intraperitoneally with the same dose of citrate-sodium citrate buffer as that of the T2DM rat. The blood was collected from the tail vein for fasting blood glucose (FBG) measurement after 72 h. FBG of 16.7 mmol/L was considered successful in T2DM model replication. The T2DM rat model was successfully replicated.

#### 2.5.2 Grouping and dosing

For better drug delivery, we used a rotary evaporator to concentrate the CR-PLR decoction, CR decoction and PLR decotion to crude drug (1 g/mL), respectively. The T2DM rats were randomly divided into 5 groups (n = 10), namely, model control group, CR (3 g of CR/kg) group, PLR (3 g of PLR/kg) group, CR-PLR (3 g of CR-PLR/kg) group, and the positive metformin group (76.5 mg/kg) group, and the drug was administered to each treatment group by gavage on the 2nd day of successful modelling in proportion to their body weights, and the model group and normal group were treated by equal amount of saline by gavage once daily for 6 weeks.

#### 2.5.3 Detection of serological indices in rats

Blood was collected from rats 6 weeks after drug administration, and the ELISA kit was used to detect FINS, HDL-C, TC, TG, TNF-α, IL-1β, IL-6, LPS, and PYY, and to calculate the insulin resistance index.

$$\text{HOMA}-\text{IR}=\frac{\text{FINS}\times \text{FBG}}{22.5}$$

#### 2.5.4 Effect of CR-PLR on the gut flora of rats

16s rDNA sequencing was used to detect changes in the gut flora of rats. The 16s rDNA gene is the DNA sequence corresponding to the ribosomal RNA encoded by bacteria, which contains 9 highly variable regions and 10 conserved regions. 16s rDNA reflects species differences and conserved regions reflect species affinity. The highly variable regions reflect interspecies differences and the conserved regions reflect interspecies affinities, and are found in the genomes of prokaryotes. 16s rDNA has a high degree of specificity, conservatism and stability. The mutation rate is low, which is suitable for microbial phylogeny and identification of indicators. 16s rRNA coding sequence is 16s rDNA, but in the experiment RNA extraction is relatively difficult, while DNA extraction is relatively easy, so this experiment on the 16s rDNA sequencing analysis. The basic principle of 16s sequencing is PCR amplification. 16s rDNA gene detection technology has become a powerful tool for microbial detection and identification.

#### 2.5.5 Effect of CR-PLR on the content of SCFAs in rats

Fresh faeces from donor rats were collected and immediately mixed with ultrapure water (1 mL). The mixture was vortexed vigorously for 3 min using a benchtop vortex and centrifuged at 12,000 rpm for 10 min. Then take 500 μL of the supernatant, add 50% sulfuric acid 50 μL, then add 550 μL of the internal standard solution (ether solution of 2-methylpentanoic acid, 100 μg/mL). The mixture was vortexed vigorously for 3 min using a benchtop vortex and centrifuged at 12,000 rpm for 10 min. Then leave it in the refrigerator (4°C) for 30 min, and then the upper layer of the ether was taken as the sample solution for analysis. We used GC-MS method to determine the content of SCFAs.

#### 2.5.6 Effect of CR-PLR on GLP-1 content in T2DM rats

We used ELISA kits to detect changes in serum GLP-1 levels in rats.

#### 2.5.7 Effect of CR-PLR on the colonic GPR43/GLP-1 pathway in T2DM rats

The expression of GPR43 and GLP-1 proteins in rat colon tissues was detected by Western blot.

The protein expression of GLP-1 and GPR43 was measured by Western blotting. In brief, the concentration of total proteins extracted from colon tissues was determined using a protein assay kit. After that, the proteins were separated by 10% SDS‒PAGE and electrotransferred to polyvinylidene fluoride membranes. The membranes were incubated with TBS-1% Tween (TBST) containing 5% skim milk powder for 2 h. The membranes were then incubated overnight at 4°C with anti-GPR43 and GLP-1 in TBST containing 5% skim milk powder. The cell membranes were then incubated with the corresponding secondary antibodies at room temperature for 1.5 h. Finally, the strips were exposed using a chemiluminescence instrument and images were captured. Data were analyzed using ImageJ software for gray value analysis.

### 2.6 Pharmacokinetics

#### 2.6.1 1Establishment of rat model of T2DM

The method is the same as “2.5.1.”

#### 2.6.2 Grouping and dosing

Eighteen normal rats were randomly divided into three groups (n = 6), CR group (7.5 g crude CR/kg), PLR group (7.5 g crude PLR/kg) and CR-PLR group (7.5 g crude CR-PLR/kg). Eighteen T2DM model rats were randomly divided into three groups (n = 6), CR group (7.5 g crude CR/kg), PLR group (7.5 g crude PLR/kg) and CR-PLR group (7.5 g crude CR-PLR/kg).

#### 2.6.3 Determination of blood concentration of rats

Prior to the experiment, the rats should be fasted for 12 h, but allowed to drink water freely. At the time points of 5 min, 10 min, 20 min, 30 min, 60 min, 120 min, 240 min, 360 min, 480 min, and 720 min of the rat’s gavage administration, 0.5 mL of blood was collected from the orbital vein and preserved in sodium heparin tubes. The samples were analyzed by LC-MS. The main pharmacokinetic parameters were calculated using DAS 3.0.

### 2.7 Cytological experiments

#### 2.7.1 Cell culture

Caco-2 cells (cat. No. CL-0050), and HT29 cells (cat. No. CL-0118) were purchased from Procell Life Science&Technology Co., Ltd. (China). Caco-2 cells were cultured in DMEM medium containing 10% fetal bovine serum, 1% non-essential amino acids, and 1% penicillin-streptomycin at a humidified atmosphere of 5% CO2 and 95% air at 37°C. HT29 cells were grown in McCoy’s 5A medium containing 10% fetal bovine serum, 1% non-essential amino acids, and 1% penicillin-streptomycin, and other conditions were the same as those for Caco-2 cells culture, with the medium being changed every other day.

#### 2.7.2 Establishment of the Caco-2/HT29 co-culture cell model

Caco-2 and HT29 cells were well-grown and mixed in a ratio of 75:25 and then inoculated at a density of 1 × 10^5^ cells/mL into 12-well plates equipped with Transwell chambers. This inoculation ratio was selected to mimic the proportion between the primary cell types in the intestine (Griesser et al., 2018). The culture conditions were consistent with those used for establishing the Caco-2 cell model. The medium was changed every 2 days during the first week and then daily from the second week onwards.

#### 2.7.3 Validation of the Caco-2/HT29 co-culture cell model

The necessity for co-cultures arises because *in vitro* cell models based on single cell lines may not accurately represent human intestinal epithelial cells. However, cell monolayers formed under varying conditions can exhibit some variability. To ensure that the cell models used in experiments closely simulate the actual conditions of intestinal epithelial cells, it is imperative to evaluate the established models against relevant criteria. In this study, we assessed the success of our cell model establishment using several indicators, including transmembrane electrical resistance (TEER) of the epithelial cells, cell polarity, and mucus staining.

##### 2.7.3.1 TEER measurement

During cell modeling, the transmembrane electrical resistance (TEER) values of the two distinct cell models must be measured periodically to assess the integrity of the cultured monolayer (Cai et al., 2022). Caco-2/HT29 co-cultured cells were inoculated into Transwell chambers, and their TEER values were measured using a Millicell ERS cytoresistometer at days 1, 4, 7, 14, and 21 of cell growth, with changes being monitored. For each well, three readings were recorded. The actual TEER values were calculated using the following formula:

$$\text{TEER}=\left(R_{t}-R_{0}\right)\times A$$

R_t_ represents the value for the experimental group; R_0_ represents the value for the blank control group; A is the area of the membrane (1.12 cm^2^).

##### 2.7.3.2 Cell polarity research

Alkaline phosphatase (ALP) activity in cell monolayers at various time points can be used to characterize cell differentiation and polarity (Calhau et al., 2002a). After inoculating the cells into 12-well Transwell chambers, the cell monolayers were gently washed with Hank’s Balanced Salt Solution (HBSS) three times. The final wash was followed by incubation at 37°C for 20 min on the 4th, 8th, 12th, 16th, and 21st day of cell growth. Subsequently, the viability of the cell monolayers on the apical (AP) and basolateral (BL) sides was determined using an ALP assay kit, following the manufacturer’s instructions. The ratio of ALP activity on the AP side to that on the BL side was used to quantify the viability of the ALP.

##### 2.7.3.3 Alcian blue staining

Compared to the Caco-2 cell model alone, the Caco-2/HT29 co-culture cell model is distinguished by its ability to produce mucus (Pan et al., 2015). The presence of mucus, as indicated by staining, is a critical determinant of whether the Caco-2/HT29 co-culture cell model has been successfully established. Caco-2/HT29 cells were inoculated into 12-well plates. On day 21 of culture, the samples were fixed with a 4% paraformaldehyde solution for 30 min. The samples were then acidified with Alcian Blue acidifying solution for 10 min, followed by the addition of Alcian Blue staining solution. The staining solution was incubated with the cells for 30 min at room temperature. After the excess stain was washed away with PBS, the membranes were carefully removed from the inserts using tweezers. The polycarbonate membranes, now containing the stained cells, were mounted on slides and examined under a Leica DMI400 B inverted fluorescence microscope.

#### 2.7.4 Cellular activity study

Cell viability was assessed using the CCK-8 assay for both CA and PUR. Caco-2 and HT29 cells were inoculated into 96-well plates at a density of 1 × 105 cells/mL. They were incubated at 37°C for 24 h to allow cell adhesion. The original medium was then replaced with 100 μL of fresh medium containing varying concentrations of CA and PUR, and the cells were further incubated for 24 h. After this, the medium was removed and the cells were co-incubated with 10 μL of CCK-8 solution for 4 h. The absorbance values (OD) were measured at 450 nm using a SpectraMax 190 Enzyme Marker. The blank group consisted of wells that were not inoculated with cells, and the control group included wells containing cells but not exposed to the sample solution. Each group was performed in six replicates. Cell viability was calculated using the following formula:

$$C\text{ell activity}\left(\%\right)=\frac{\left(\mathrm{Exp}erimental\;\text{Group}\;OD\;value-Blank\;\text{Group}\;\text{OD}\;v\text{alue}\right)}{\left(C\text{ontrol}\;\text{Group}\;\text{OD}\;\text{value}-Blank\;\text{Group}\;\text{OD}\;v\text{alue}\right)}\times 100\%$$

#### 2.7.5 Bidirectional transport of PUR: Effects of CA co-administration

Before conducting the experiment, a Caco-2/HT29 co-culture cell monolayer model that met the criteria for transepithelial transport was selected. The drug delivery solutions, including geranylgeranyl and a combination of CA and geranylgeranyl, were prepared using Hank’s Balanced Salt Solution (HBSS). These solutions were then filtered through a 0.22 μm membrane to eliminate any bacterial contamination, after which they were ready for use. Subsequently, transmembrane transport studies of the drugs were carried out using the Caco-2/HT29 co-culture cell model. HPLC was utilized to ascertain the drug content. The apparent permeability coefficient *P*_*app*_ (cm/s) (Saez-Tenorio et al., 2019) and the efflux ratio (ER) (Kobayashi et al., 2012) were calculated using the subsequent equations:

$$P_{app}=\frac{d_{Q}\times d_{t}}{A\times C_{0}}$$

$$ER=\frac{P_{app}\left(B-A\right)}{P_{app}\left(A-B\right)}$$

A: Represents the area of the permeable membrane, which is 1.12 cm^2^ C_0_: Represents the initial drug concentration in the supply pool, measured in micrograms per milliliter (μg/mL). d_Q_/d_t_: Represents the diffusive flux, which can be determined from the slope of a straight line obtained by performing a linear regression fit to the cumulative drug transit versus time curve.

#### 2.7.6 Effects of efflux transporter inhibitors on the transport of PUR

Many poorly absorbed oral drugs are affected by cellular efflux, with P-glycoprotein (P-gp), Breast Cancer Resistance Protein (BCRP), and Multidrug Resistance-Associated Protein 2 (MRP2) being the most representative efflux transporter proteins in the intestine (Li et al., 2018; Ming et al., 2011; Kikuchi et al., 2022). To explore the relationship between the cellular transport of Pur and the inhibition of these efflux proteins, this study incorporated the use of three transporter protein inhibitors: Verapamil (Ver), Ko143, and MK-571.

### 2.8 Statistical analysis

Statistical analysis was performed using the Origin 2019b software (Origin Lab, Northampton, MA) and expressed as the mean ± standard deviation (SD). One-way analysis of variance (ANOVA) was used to conduct statistical analyze of the results. Any value of *P* < 0.05 was considered to be significant. *P* < 0.01 was considered very significant, and *P* < 0.001 was extremely significant.

## 3 Results

### 3.1 Compositional analysis of CR-PLR decoction

#### 3.1.1 Analysis of CR-PLR decoction by UPLC-Q-TOF-MS

**FIGURE 1:**
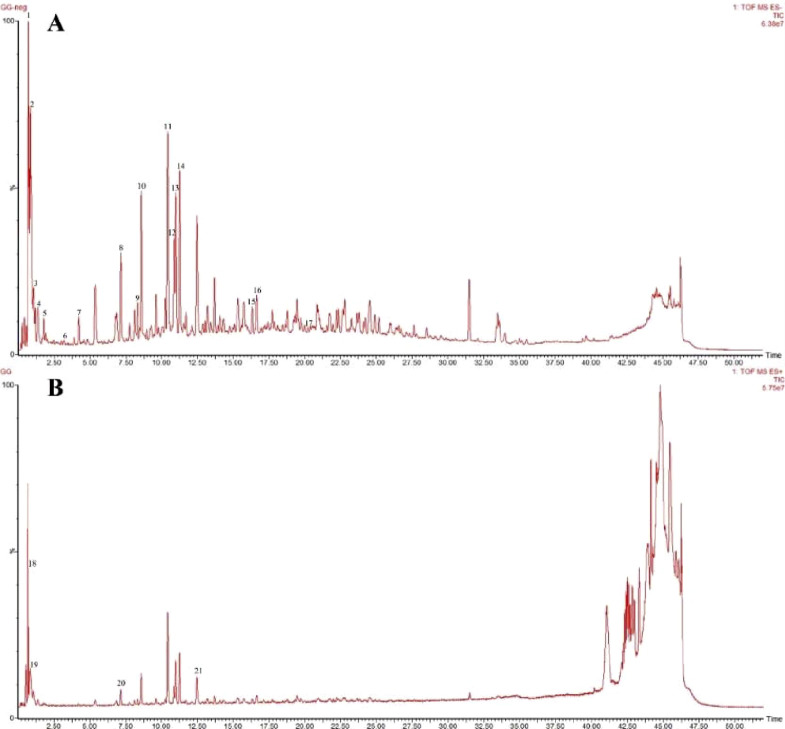
Negative ion pattern **(A)** and positive ion pattern **(B)** of the aqueous extract of CR-PLR.

A total of 21 components were analyzed in the CR-PLR decoction in positive and negative ion mode, among which 11 components derived from CR and 10 components derived from PLR. The results were shown in Figure 1 and Table 1.

**TABLE 1:** UPLC-Q-TOF-MS analysis of CR-PLR decoction.

No.	Retention time/min	Molecule	Theoretical molecular weight (m/z)	Measured molecular weight (m/z)	Mode	Name	Secondary fragment ions (m/z)	Source
1	0.68	C_10_H_8_O_2_	159.0446	159.0289	Negative	6-Methylcoumarin	145.0126, 117.0196	CR
2	0.82	C_10_H_8_O_3_	175.0395	175.0641	Negative	7-Methoxycoumarin	161.0432	CR
3	1.06	C_9_H_10_O	133.0653	133.0139	Negative	Cinnamyl alcohol	115.0033	CR
4	1.46	C_9_H_6_O_4_	177.0188	177.0383	Negative	6,7-Dihydroxycoumarin	149.0088	CR
5	1.67	C_7_H_6_O_5_	169.0137	169.0141	Negative	Gallic acid	125.0235	CR
6	3.20	C_10_H_12_O_2_	163.0759	163.0382	Negative	Eugenol	133.0139	CR
7	4.22	C_9_H_8_O_4_	179.0344	179.0303	Negative	Caffeic acid	161.0382	PLR
8	7.16	C_27_H_30_O_14_	577.1557	577.1541	Negative	Puerarin-4′-O-β-D-glucopyranoside	457.1148	PLR
9	8.33	C_7_H_6_O_4_	153.0188	153.0191	Negative	3,4-Dihydroxybenzoic acid	109.0290	CR
10	8.59	C_21_H_20_O_10_	431.0978	431.0922	Negative	Genistein-8-c-glycoside	269.0461, 151.0399	PLR
11	10.44	C_21_H_20_O_9_	415.1029	415.1112	Negative	Puerarin	397.2246, 379.0806, 361.1142, 349.0616, 319.1541, 295.0581, 277.0329 267.0648	PLR
12	10.91	C_9_H_8_O_2_	147.0446	147.0446	Negative	Cinnamic acid	129.0536, 101.0592	CR
13	11.06	C_22_H_22_O_10_	445.1135	445.1101	Negative	3′-Methoxypuerarin	223.0446, 308.0649	PLR
14	11.26	C_26_H_28_O_13_	547.1452	547.1494	Negative	Puerarin apioside	486.3486, 293.1779	PLR
15	16.29	C_22_H_22_O_9_	429.1186	429.1133	Negative	4′-Methoxypuerarin	267.0648, 307.0596	PLR
16	16.65	C_15_H_10_O_4_	253.0501	253.0455	Negative	Daidzein	224.0479, 208.0687	PLR
17	20.23	C_20_H_22_O_6_	357.1338	357.1012	Negative	Pinoresinol	339.2005, 309.1718, 203.0206	CR
18	0.78	C_9_H_8_O	133.0653	133.0626	Positive	Cinnamaldehyde	105.1120	CR
19	0.87	C_7_H_6_O_3_	139.0395	139.0387	Positive	Protocatechuic aldehyde	121.0311	CR
20	7.16	C_27_H_30_O_14_	579.1714	579.1715	Positive	Daidzein-4′,7-diglucoside	338.1171, 205.0915	PLR
21	12.47	C_21_H_20_O_9_	417.1186	417.1209	Positive	Daidzin	255.0461, 132.9011	PLR

#### 3.1.2 HPLC analysis of CR-PLR decoction

**FIGURE 2:**
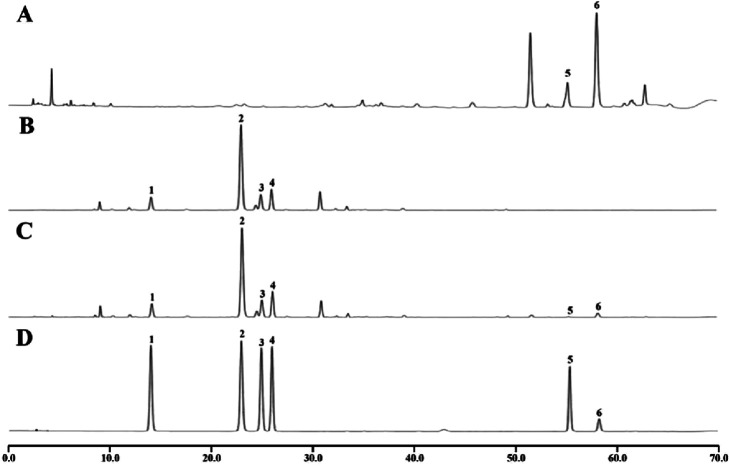
HPLC analysis of the test and reference substance, CR decoction **(A)**; PLR decoction **(B)**; CR-PLR decoction **(C)**; reference substance **(D)** (1: 3′-hydroxy puerarin; 2: Puerarin; 3: 3′- Methoxy puerarin; 4: Puerarin apioside; 5: Cinnamic acid; 6: Cinnamaldehyde).

From Figure 2 and Table 2, it can be seen that CA and cinnamaldehyde are derived from CR. 3′-hydroxypuerarin, puerarin, 3′- methoxypuerarin amd puerarin apioside are derived from PLR. Meanwhile, we found that the content of cinnamaldehyde was 2.48 times higher than that of CA in the CR decoction. In the PLR decoction, the content of puerarin (41.465 mg/g) was significantly higher than that of 3′- Methoxypuerarin (9.009 mg/g), Puerarin apioside (12.044 mg/g) and 3′-hydroxy puerarin (7.372 mg/g). However the CR-PLR decoction also had the highest content of puerarin (40.920 mg/g).

**TABLE 2:** Content of the substance to be measured in the decoction.

Substance	CR decoction (mg/g)	PLR decoction (mg/g)	CR-PLR decoction (mg/g)
3′-hydroxy puerarin	\	7.372	7.375
Puerarin	\	41.465	40.920
3′- Methoxy puerarin	\	9.009	9.461
Puerarin apioside	\	12.044	13.433
Cinnamic acid	3.967	\	2.393
Cinnamaldehyde	9.823	\	10.564

### 3.2 Pharmacodynamic

#### 3.2.1 Blood glucose and weight

**FIGURE 3:**
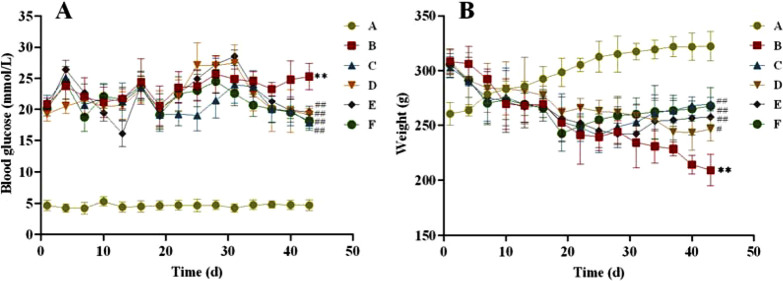
**(A)** Changes of blood glucose in rats of each group; **(B)** Changes of body weight in rats in each group. (‾x ± SD, n = 6), Comparison with normal group, **P < 0.01, Comparison with model group, ^#^ *P* < 0.05, ^##^ *P* < 0.01 (A: Normal group; B: Model group; C: Metformin group; D: CR group; E: PLR group; F: CR-PLR group).

After four weeks of administration, in terms of blood glucose (Figure 3), we found that blood glucose was relatively stable in the normal and T2DM groups of rats. However, after the intervention of CR and PLR, the blood glucose of rats were significantly decreased compared with the T2DM rats (*P* < 0.01). Among them, the hypoglycemic effect of CR-PLR group was close to that of metformin group. In terms of body weight, the weight of rats in the normal group gradually increased, the weight of rats in the model group gradually decreased, and the weight of rats in the metformin group and each group of the TCM first decreased and then increased with the increase of the administration time: the weight of the CR group increased after 6 weeks of administration; the weight of the metformin group and the PLR group increased after 4 weeks of administration; and the weight of the CR-PLR group increased after 3 weeks of administration.

#### 3.2.2 Detection of serological indices in rats

**FIGURE 4:**
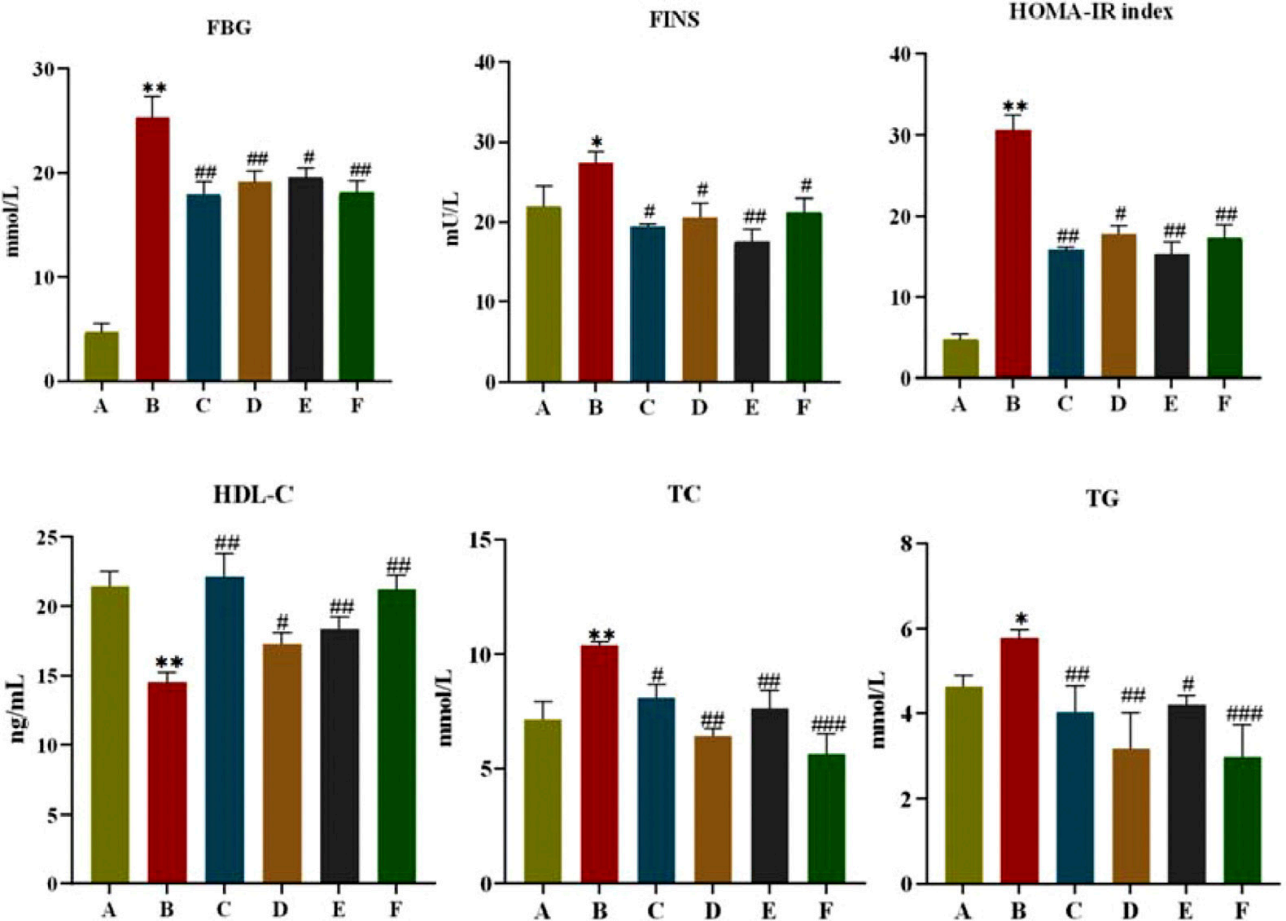
FBG, FINS, HOMA-IR index, HDL-C, TC, TG index in each group of rats, (‾x ± SD, n = 6), Comparison with normal group, *P < 0.05, **P < 0.01, Comparison with model group, ^#^ *P* < 0.05, ^##^ *P* < 0.01, ^###^ *P* < 0.01 (A: Normal group; B: Model group; C: Metformin group; D: CR group; E: PLR group; F: CR-PLR group).

**FIGURE 5:**
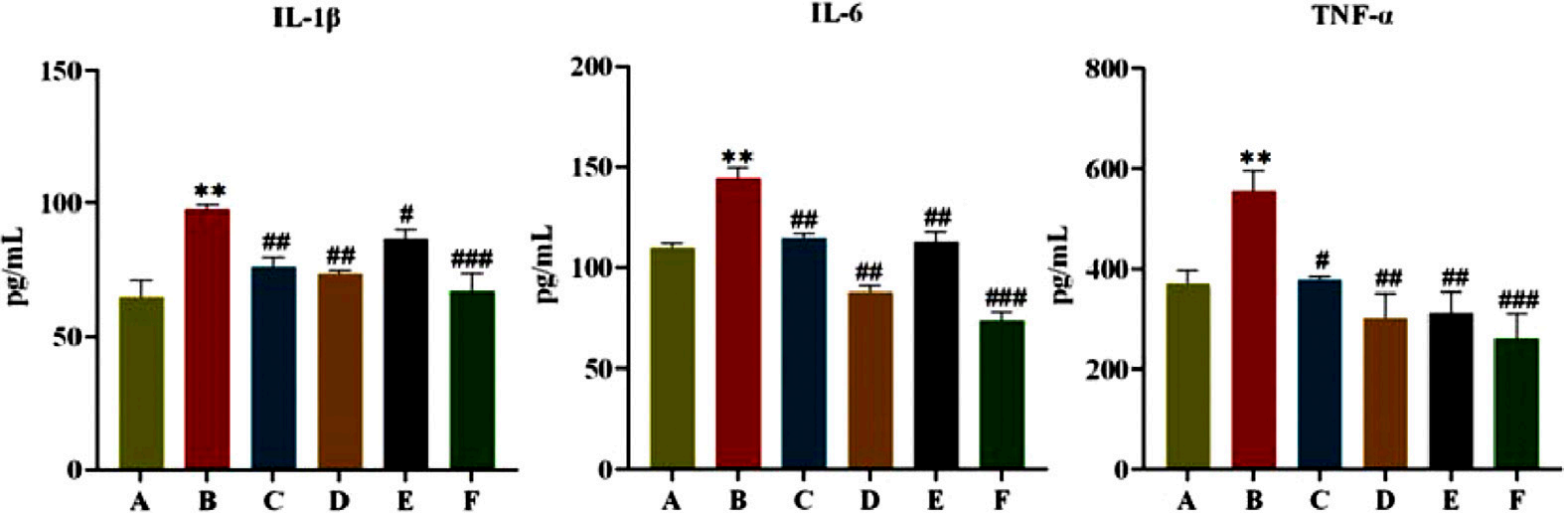
Content of IL-1β, IL-6, and TNF-α in serum in each group of rats, ( $\bar{x}$ ± SD, n = 6), Comparison with normal group, **P < 0.01, Comparison with model group, ^#^ *P* < 0.05, ^##^ *P* < 0.01, ^###^ *P* < 0.001 (A: Normal group; B: Model group; C: Metformin group; D: CR group; E: PLR group; F: CR-PLR group).

**FIGURE 6:**
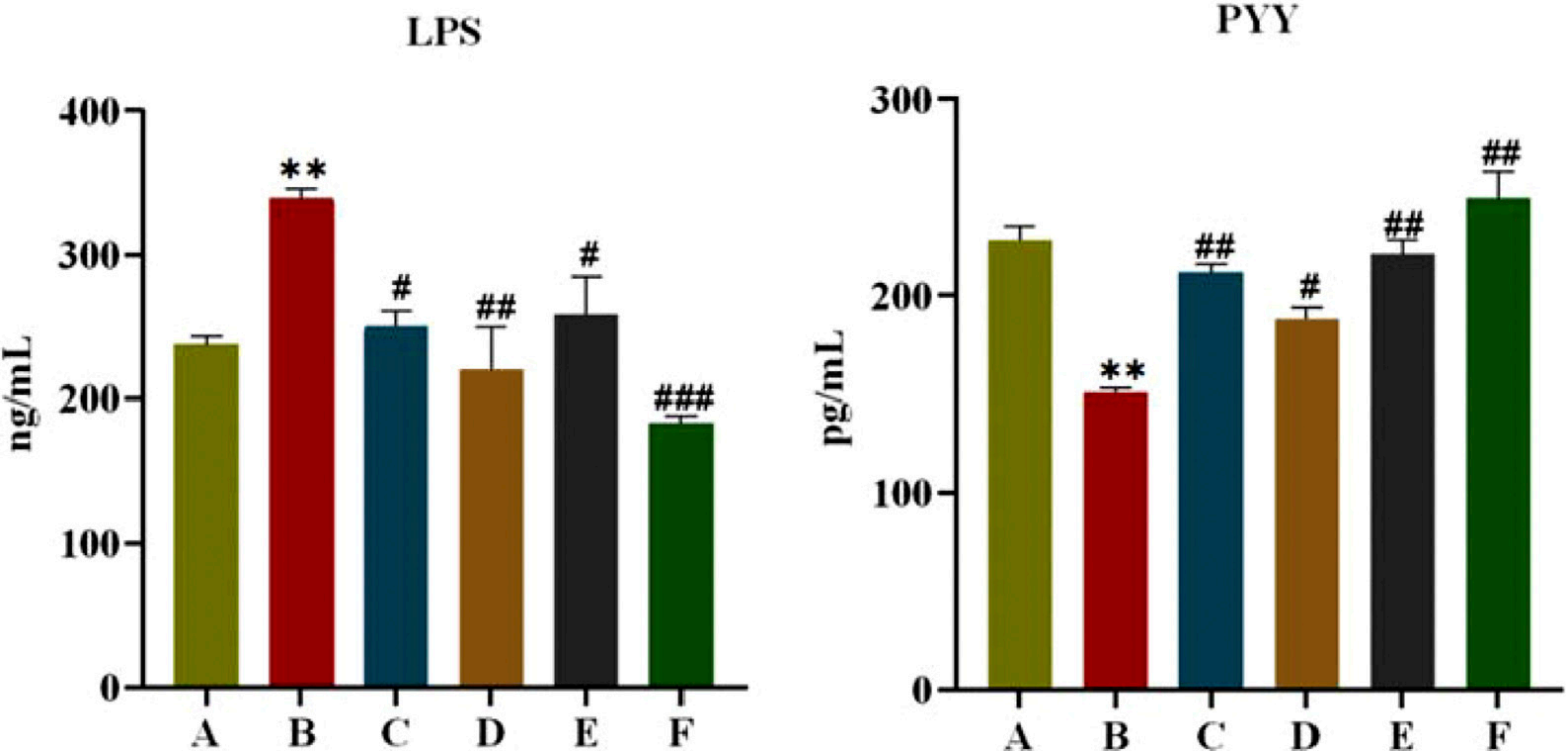
Content of LPS and PYY in serum in each group of rats, (‾x ± SD, n = 6), Comparison with normal group, *P < 0.01, Comparison with model group, ^#^ *P* < 0.05, ^##^ *P* < 0.01, ^###^ *P* < 0.01 (A: Normal group; B: Model group; C: Metformin group; D: CR group; E: PLR group; F: CR-PLR group).

As shown in Figure 4, after 6 weeks of administration, FINS and HOMA-IR of rats in the model group were significantly higher compared with the normal group (*P* < 0.05, *P* < 0.01), and FINS and HOMA-IR of rats in each treatment group were significantly lower than those in the model group (*P <* 0.05, *P* < 0.01). This indicates that CR and PLR alone or in combination can improve IR levels in T2DM rats. In terms of lipid metabolism, TC and TG levels were significantly reduced and HDL-C levels were significantly increased in all treatment groups after CR and PLR intervention (*P* < 0.05). This indicates that CR and PLR alone or in combination can improve lipid metabolism levels in T2DM rats. According to Figure 5, the levels of IL-1β, IL-6 and TNF-α were significantly higher in the model group (*P* < 0.05), and the levels of IL-1β, IL-6 and TNF-α were significantly lower in each treatment group compared with those in the model group (*P* < 0.05). This indicates that CR, PLR and CR-PLR can improve the levels of inflammatory factors in T2DM rats. In terms of improving T2DM levels, the LPS level in the model group was significantly higher than that in the normal group (*P* < 0.01), but the PYY level was significantly lower than that in the normal group (*P* < 0.01). After the intervention of CR and PLR, the LPS level was significantly reduced while the PYY level was increased with Figure 6 (*P* < 0.05). This indicates that CR, PLR and CR-PLR can reduce LPS and increase PYY levels in T2DM rats to achieve the therapeutic effect of T2DM.

#### 3.2.3 Effect of CR-PLR on the gut flora of rats

**FIGURE 7:**
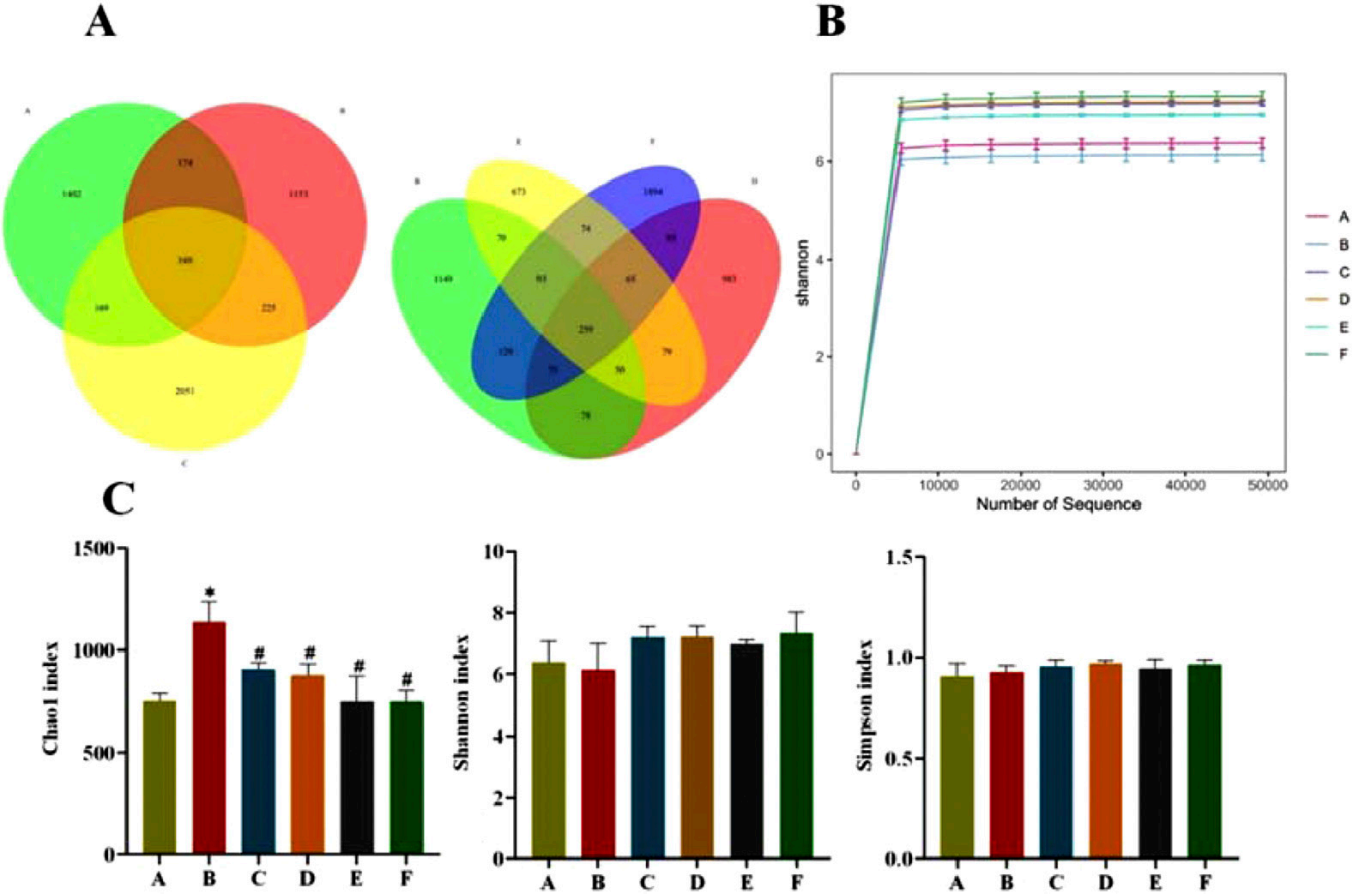
**(A)** OTU Venn diagram of gut flora; **(B)** Sample Shannon Sparse Curve; **(C)** Alpha Diversity Index (A: Normal group; B: Model group; C: Metformin group; D: CR group; E: PLR group; F: CR-PLR group) Comparison with normal group, *P < 0.01, Comparison with model group, ^#^ *P* < 0.05.

The Venn plot can provide a more intuitive representation of the species composition of environmental samples (such as OTUs) among different groups of rats. As can be seen from Figure 7A, the total number of OTUs in the normal and model groups was 522, the number of independent OTUs in the normal group was 1,402, and the number of independent OTUs in the model group was 1,153, which indicates that the gut flora of rats in the model group in the same living environment has already differed from that of rats in the normal group. The number of independent OTUs in the metformin group was 2051, and the number of OTUs that overlapped with the model group was higher than the number of OTUs that overlapped with the normal group, indicating a higher similarity in the microbial community between the metformin group and the model group. The CR group and the model group have the least number of overlapping OTUs, indicating the lowest similarity in gut microbiota between the CR group and the model group in rats.

Shannon sparse curves are shown in Figure 7B, and the end of the curves of the samples in each group were gradually smooth, which can indicate that the sequences in each group have reached saturation, and the sequencing amount of the samples in each group can reflect the diversity of the gut flora. According to Figure 7C, compared with group A, the species richness (Chao1 index) in the faeces of T2DM rats in group B was significantly higher (*P* < 0.05), whereas there was basically no difference in the species diversity (Shannon and Simpson indices) in each group.

**FIGURE 8:**
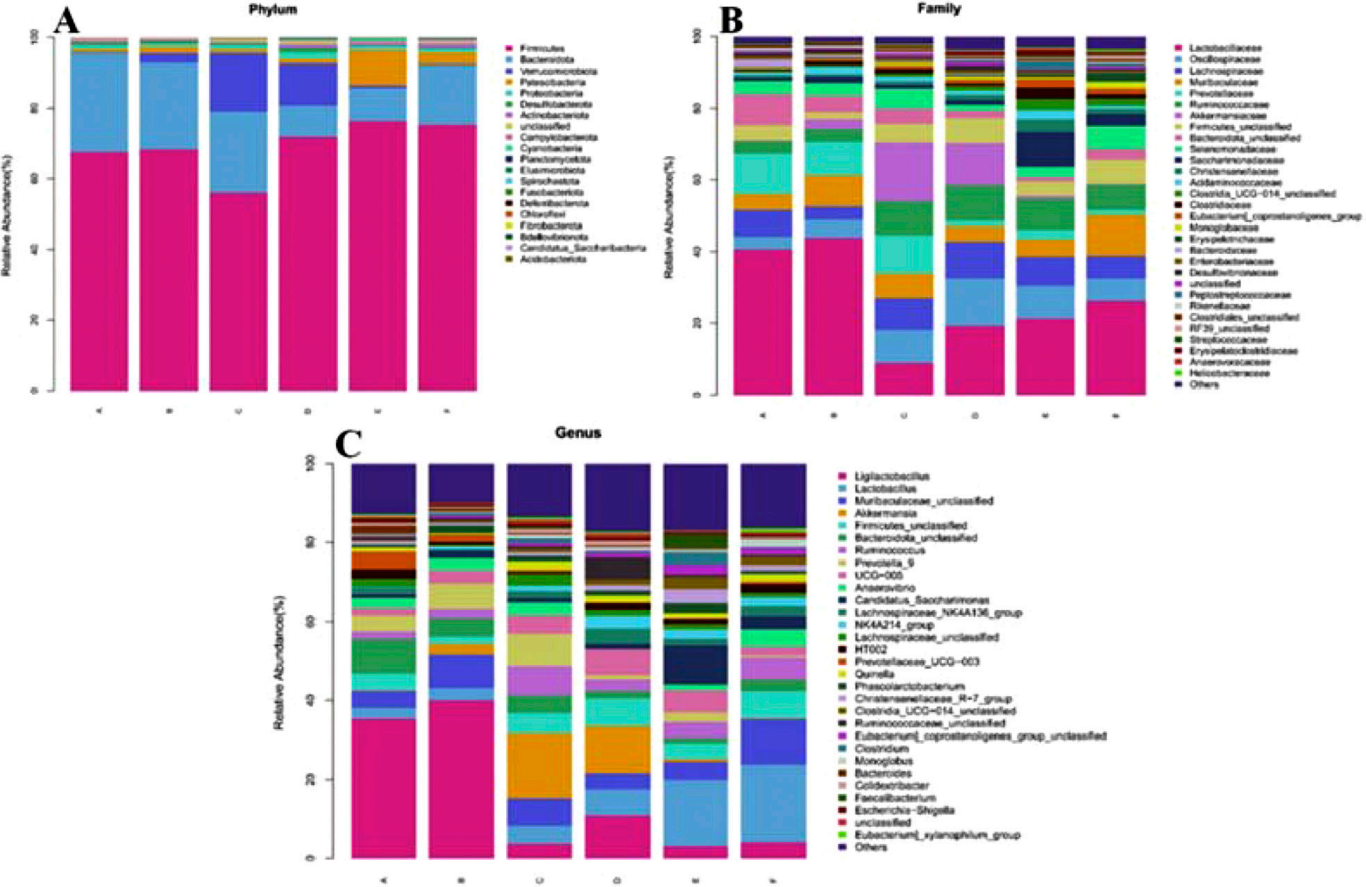
**(A)** Analysis of community composition at the Phylum level; **(B)** Analysis of community composition at the Family level; **(C)** Analysis of community composition at Genus level (A: Normal group; B: Model group; C: Metformin group; D: CR group; E: PLR group; F: CR-PLR group).

The community composition was analyzed at the level of Phylum (Figure 8A): most of them were distributed in Firmicutes, Bacteroidota, Verrucomicrobiota and Patescibacteria. On the one hand, the relative abundance of Bacteroidota in the feces of rats in the model group (68.33%) was higher than the normal group (67.60%). However, the relative abundance of Bacteroidota in the feces of rats in the model group (24.48%) was lower than in the normal group (27.98%). After the intervention of CR and PLR (CR group: 71.89%, PLR group: 76.36%, CR-PLR group: 75.30%), the relative abundance of Firmicutes was significantly higher than the model group, while the relative abundance of Bacteroidota was significantly lower. On the other hand, the relative abundance of Verrucomicrobiota in the CR group (11.83%) was significantly increased compared with the model group; and the relative abundance of Patescibacteria in the PLR group (9.95%) and CR-PLR group (3.33%) was significantly increased compared with the model group.

Community composition analysis at the family (Family) level (Figure 8B): the percentage of Lactobacillaceae flora was reduced in each treatment group, which was 40.61% in the normal group, 43.82% in the model group, 8.88% in the metformin group, 19.19% in the CR group, 21.32% in the PLR group, and 26.21% in the CR-PLR group. The percentage of Lachnospiraceae flora was increased in each treatment group compared to the model group, which was 7.28% in the normal group, 3.68% in the model group, 8.91% in the metformin group, 10.22% in the CR group, 8.11% in the PLR group, and 6.27% in the CR-PLR group. Moreover, each treatment group increased the percentage of the Ruminococcaceae flora, which was 3.49% in the normal group, 3.54% in the model group, 9.47% in the metformin group, 9.91% in the CR group, 8.70% in the PLR group, and 6.94% in the CR-PLR group. Lachnospiraceae and Ruminococcaceae are usually anaerobic bacteria belonging to the group of Firmicutes, and the flora of these two families have been reported to be associated with the production of SCFAs (Ma et al., 2020). In addition, the flora of Lachnospiraceae are producers of butyrat (Li B. et al., 2021; McCulloch et al., 2022).

Analysis of community composition at Genus level: As shown in Figure 8C, the percentage of Ligilactobacillus flora was significantly decreased in all treatment groups compared to the model group, which was 40.06% in the model group, 3.69% in the metformin group, 10.95% in the CR group, 3.13% in the PLR group, 3.99% in the CR-PLR group. On the contrary, the percentage of lactobacilli flora increased, 3.08% in the model group, 4.68% in the metformin group, 6.54% in the CR group, 16.76% in the PLR group and 19.89% in the CR-PLR group. The percentage of butyrate-producing bacterial Akkermansia and Ruminococcus flora increased in the metformin and CR groups compared to the model group. Whereas, the percentage of Ruminococcus flora increased in the PLR group and the CR-PLR groups compared to the model group.

#### 3.2.4 Effect of CR-PLR on the content of SCFAs in rats

**FIGURE 9:**
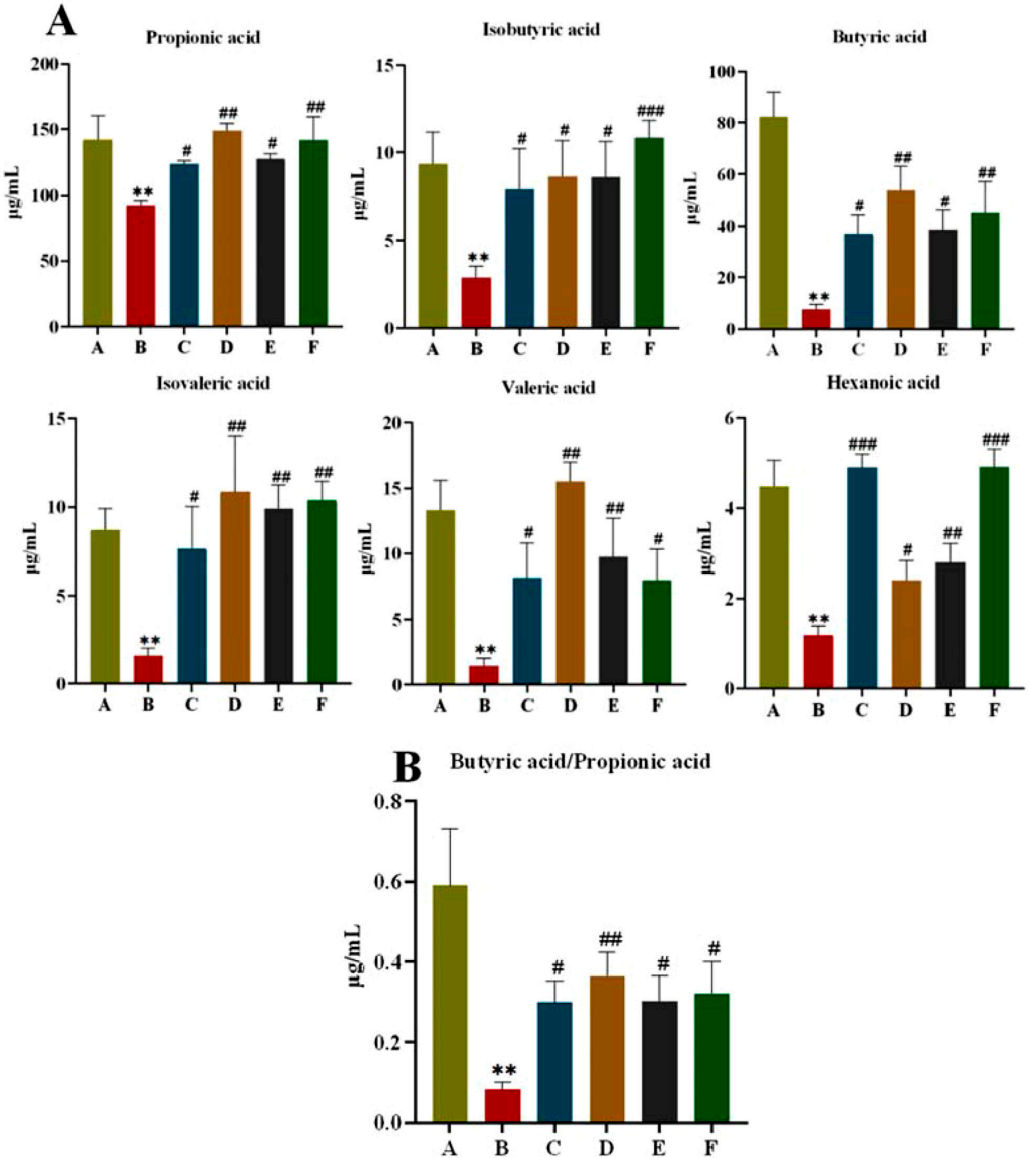
**(A)** Content of short-chain fatty acids in each group of rats; **(B)** Ratio of butyric acid to propionic acid in each group of rats, Comparison with normal group, **P < 0.01, Comparison with model group, ^#^ *P* < 0.05, ^##^ *P* < 0.01 (A: Normal group; B: Model group; C: Metformin group; D: CR group; E: PLR group; F: CR-PLR group).

SCFAs are fermentation products of intestinal bacteria. As shown in Figure 9A, the SCFAs of rats in the model group were significantly lower (*P* < 0.01) compared with those in the normal group; and the SCFAs of rats in all treatment groups were significantly higher (*P* < 0.01, *P* < 0.05) compared with those in the model group. Meanwhile, we found that the CR-PLR group was superior to the metformin group in increasing Propionic acid, Isobutyric acid, Butyric acid, and Isovaleric acid. The butyric acid/propionic acid ratio was significantly lower in the model group compared with the normal group (*P* < 0.01); and the butyric acid/propionic acid ratio was significantly higher in all treatment groups compared with the model group (Figure 9B). This suggests that CR, PLR and CR-PLR can increase the levels of SCFAs in T2DM rats, which may be a potential mechanism for the improvement of IR by CR-PLR.

#### 3.2.5 Effect of CR-PLR on GLP-1 content in T2DM rats

**FIGURE 10:**
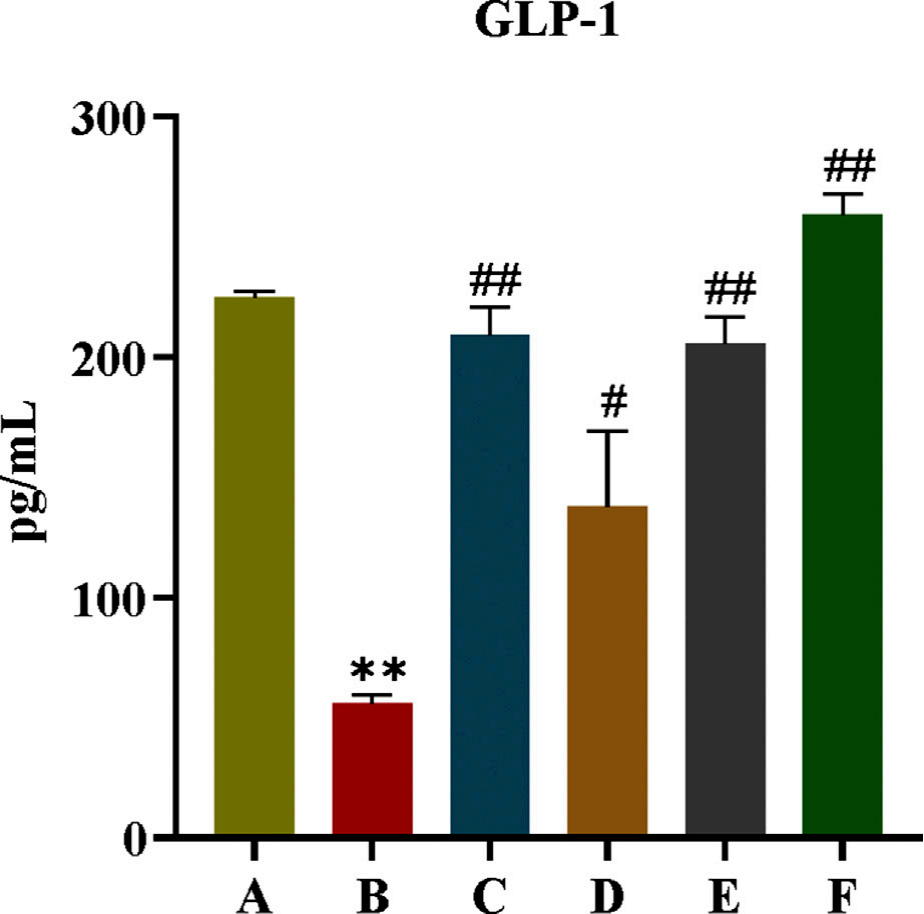
GLP-1 content in each group of rats, Comparison with normal group, **P < 0.01, Comparison with model group, ^#^ *P* < 0.05, ^##^ *P* < 0.01 (A: Normal group; B: Model group; C: Metformin group; D: CR group; E: PLR group; F: CR-PLR group).

GLP-1 is an endogenous hypoglycaemic hormone secreted by intestinal L cells and has an important role in T2DM. As can be seen from Figure 10, compared with rats in the normal group, the content of GLP-1 in the model group was significantly reduced (*P* < 0.01). When we gave CR, PLR and CR-PLR interventions, the GLP-1 content of rats in each treatment group was significantly elevated (*P* < 0.01). Interestingly, the GLP-1 content of rats in the CR-PLR group was even higher than that of rats in the normal group. This suggests that CR and PLR can increase the level of GLP-1 in T2DM rats, and the effect of CR-PLR group is stronger than that of metformin group, which may be a potential mechanism of CR-PLR to improve IR.

#### 3.2.6 Effect of CR-PLR on the colonic GPR43/GLP-1 pathway in T2DM rats

**FIGURE 11:**
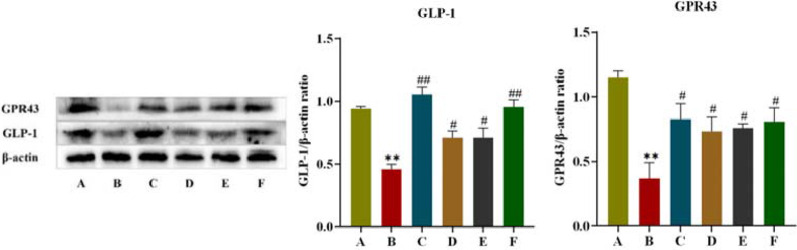
Expression of GPR43 and GLP-1 proteins in colon tissues in each group of rats, Comparison with normal group, **P < 0.01, Comparison with model group, ^#^ *P* < 0.05, ^##^ *P* < 0.01 (A: Normal group; B: Model group; C: Metformin group; D: CR group; E: PLR group; F: CR-PLR group).

GPR43 is one of the G protein-coupled receptors in the intestine, which mediates SCFAS to promote the secretory effects of GLP-1, and GPR43 also promotes energy metabolism in tissue cells. In this experiment, the effect of CR-PLR on the GPR43/GLP-1 pathway in rat colon was further investigated in terms of protein expression. As shown in Figure 11, compared with rats in the normal group, both colonic GPR43 and GLP-1 protein expression were significantly reduced in the model group; compared with rats in the model group, both GPR43 and GLP-1 protein expression were significantly elevated in rats in each administration group. The results indicated that both CR and PLR alone or in combination could upregulate the GPR43/GLP-1 pathway in the colon of T2DM rats and exert the effect of improving glucolipid metabolism and IR in T2DM rats.

## 4 Pharmacokinetics

**FIGURE 12:**
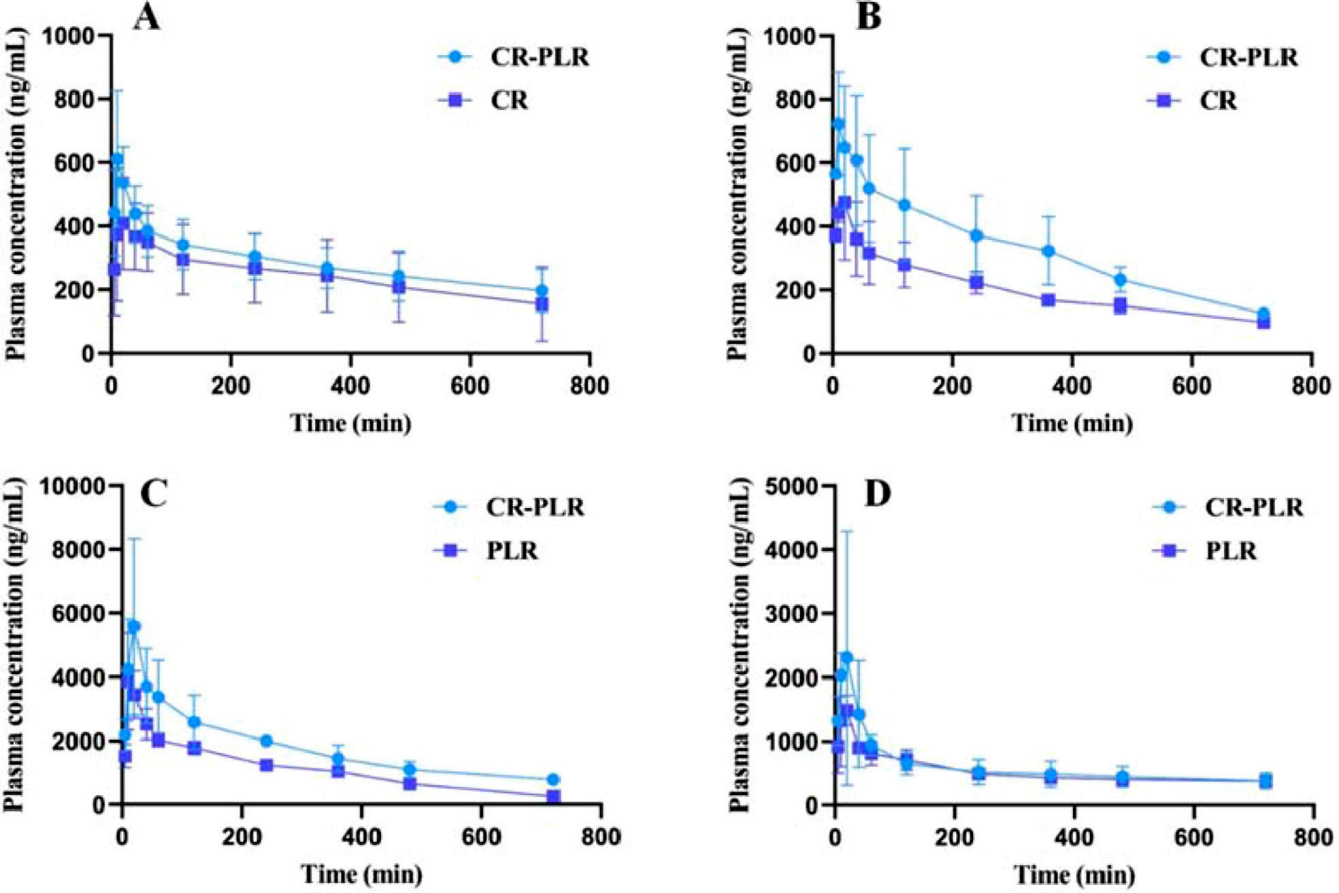
**(A)** Cinnamic acid plasma concentration-time curve in the normal group; **(B)** Cinnamic acid plasma concentration-time curve in the model group; **(C)** Puerarin plasma concentration-time curve in the normal group; **(D)** Puerarin plasma concentration-time curve in the model group (‾x ± SD,n = 6)

For the normal group of rats, the plasma concentration-time curve of CA in the CR-PLR and CR groups are shown in Figure 12A, and the plasma pharmacokinetic parameters are shown in Table 3. The plasma concentration-time curve of puerarin in the CR-PLR and PLR groups are shown in Figure 12B, and the plasma pharmacokinetic parameters are shown in Table 4. Based on PUR, we found that the plasma concentration in the CR-PLR group was significantly higher than that of the PLR group. In the PLR group, the plasma concentration peaked after 12 min, and the C_max_ at the peak was 4,241.59 ± 1,084.43 μg/L, which was close to the 10 min plasma concentration in the CR-PLR group (4,218.74 ± 1,428.58 μg/L). In the CR-PLR group, the peak time was 16 min and the peak plasma concentration C_max_ was 6,002.78 ± 2,285.85 μg/L. This indicates that CR-PLR can reach the required plasma concentration in a shorter period of time in rats and enable more drugs to enter the blood circulation. In addition, the C_max_, AUC_0-12_, AUC_0-∞_, and MRT of the CR-PLR group were 1.42, 1.59, 1.93, and 1.10 times higher than those of the PLR group, respectively. Meanwhile, the relative bioavailability increased by 159.23%. This suggests that CR-PLR versus CR not only allowed more drugs of geranylgeranyl to enter the blood circulation, but also stayed longer in the rats. As for CA, there was no significant difference between the plasma concentration in the CR-PLR group and the CR group.

**TABLE 3:** Plasma pharmacokinetic parameters of cinnamic acid after oral administration of CR-PLR granules and CR granules in the normal group of rats (‾x ± SD, n = 6).

Parameters	CR-PLR	CR
C_max_ (μg/L)	649.17 ± 164.08	447.39 ± 147.28
T_max_ (min)	12.00 ± 4.00	18.00 ± 4.00
t_1/2_ (min)	1,079.43 ± 786.59	771.40 ± 634.44
AUC_0-12_ (μg/L·min)	206,019.72 ± 43,644.83	175,519.40 ± 69,730.96
AUC_0-∞_ (μg/L·min)	377,346.55 ± 121,133.54	240,789.33 ± 66,280.28
MRT_0-t_ (min)	306.40 ± 19.64	301.68 ± 24.42

**TABLE 4:** Plasma pharmacokinetic parameters of puerarin after oral administration of CR-PLR granules and PLR granules in the normal group of rats (‾x ± SD, n = 6).

Parameters	CR-PLR	PLR
C_max_ (μg/L)	6,002.78 ± 2,285.85	4,241.59 ± 1,084.43
T_max_ (min)	16.00 ± 4.90	12.00 ± 4.00
t_1/2_ (min)	394.06 ± 40.01	217.42 ± 44.69*
AUC_0-12_ (μg/L·min)	1,275,552.32 ± 141,773.27	801,072.80 ± 60,592.92*
AUC_0-∞_ (μg/L·min)	1,724,483.50 ± 206,055.43	891,503.08 ± 44,305.01*
MRT_0-t_ (min)	256.99 ± 31.68	233.36 ± 9.40

*P < 0.05, Indicates significant difference (CR-PLR group compared with PLR group).

For the model group rats, the plasma concentration-time curve of CA in the CR-PLR group and CR group rats are shown in Figure 12C, and the plasma pharmacokinetic parameters are shown in Table 5. The plasma concentration-time curve of puerarin in the CR-PLR and PLR groups of rats are shown in Figure 12D, and the plasma pharmacokinetic parameters are shown in Table 6. Based on puerarin, we found that after oral administration to rats, the plasma concentration in the CR-PLR group was significantly higher than that in the PLR group. After gavage administration, the plasma concentration in both CR-PLR and PLR groups reached its peak 15 min later. At the peak, the plasma concentration C_max_ in the CR-PLR group was 2,756.55 ± 951.48 μg/L, the blood drug concentration C_max_ of the PLR group is 1,578.23 ± 252.05 μg/L. In addition, the C_max_, AUC_0-12_, and AUC_0- ∞_of CR-PLR are 1.75, 1.13, and 1.71 times higher than those of PLR, respectively. This indicates that CR-PLR can reach the required plasma concentration in rats in a shorter time and enable more drugs to enter the bloodstream. For CA, there was no significant difference in plasma concentration between the CR-PLR group and the CR group.

**TABLE 5:** Plasma pharmacokinetic parameters of cinnamic acid after oral administration of CR-PLR granules and CR granules in the model group of rats (‾x ± SD, n = 6).

Parameters	CR-PLR	CR
C_max_ (μg/L)	723.28 ± 115.24	533.42 ± 70.01
T_max_ (min)	10.00	15.00 ± 5.00
t_1/2_ (min)	329.42 ± 48.07	365.65 ± 92.99
AUC_0-12_ (μg/L·min)	232,832.48 ± 47,399.51	143,032.11 ± 10,673.33
AUC_0-∞_ (μg/L·min)	292,142.24 ± 44,006.00	188,352.51 ± 6,196.84
MRT_0-t_ (min)	271.13 ± 7.89	276.78 ± 19.00

**TABLE 6:** Plasma pharmacokinetic parameters of puerarin after oral administration of CR-PLR granules and PLR granules in the model group of rats (‾x ± SD, n = 6).

Parameters	CR-PLR	PLR
C_max_ (μg/L)	2,756.55 ± 951.48	1,578.23 ± 252.05
T_max_ (min)	15.00 ± 5.00	15.00 ± 5.00
t_1/2_ (min)	990.94 ± 154.60	469.65 ± 111.16*
AUC_0-12_ (μg/L·min)	429,132.95 ± 116,848.53	381,001.35 ± 3,482.60
AUC_0-∞_ (μg/L·min)	951,618.25 ± 162,505.14	558,079.40 ± 5,039.27*
MRT_0-t_ (min)	278.97 ± 4.33	292.93 ± 9.58

*P < 0.05, Indicates significant difference (CR-PLR group compared with PLR group).

In summary, the absorption of puerarin in the CR-PLR group was better than that in the PLR group, both in the normal and model groups. And there was no significant difference in any of the CA. This suggests that some components in CR promote the absorption of puerarin and increase the drug efficacy after the combination of the two herbs.

## 5 Cell experiment results

### 5.1 TEER

**FIGURE 13:**
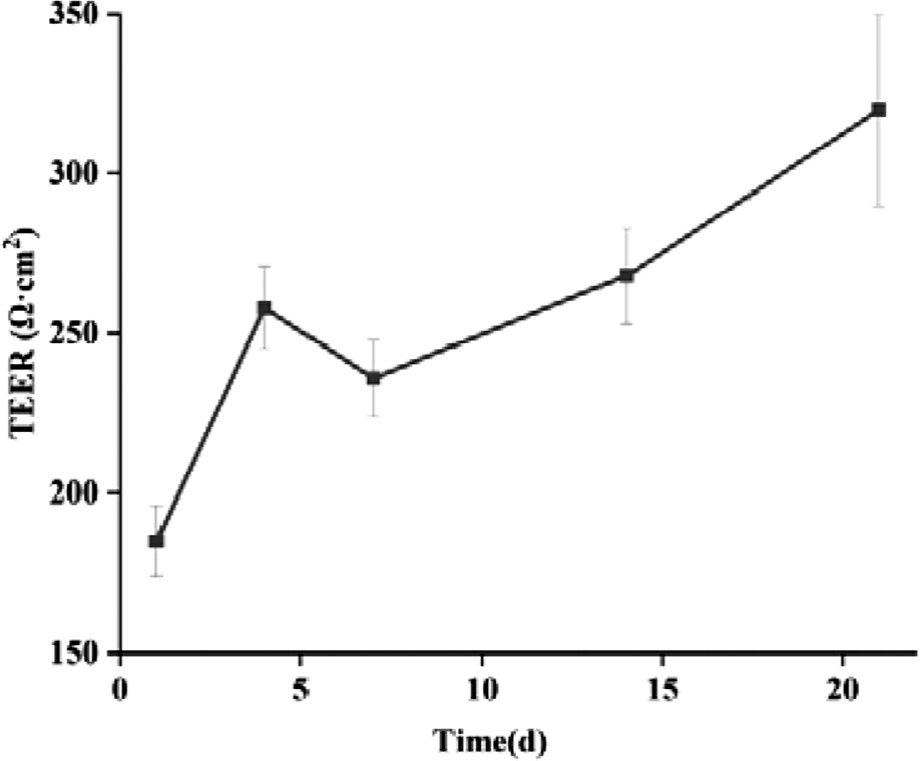
Changes of TEER values of Caco-2/HT29 co-culture cell model with culture time (‾x ± SD, n = 3).

By monitoring the TEER values in cell models, the integrity of cell fusion and cell monolayer junctions can be assessed over a 21-day period. As depicted in Figure 13, the TEER values for the Caco-2/HT29 co-culture cell model increased progressively with incubation time, ultimately reaching and surpassing the minimum TEER value criterion of 250 Ω·cm^2^ necessary for transcellular transport studies (Srinivasan et al., 2015). These findings suggest that the Caco-2/HT29 co-cultured cells form a desirable and intact epithelial layer under *in vitro* conditions, establishing a foundation for further research.

### 5.2 Cell polarity

**FIGURE 14:**
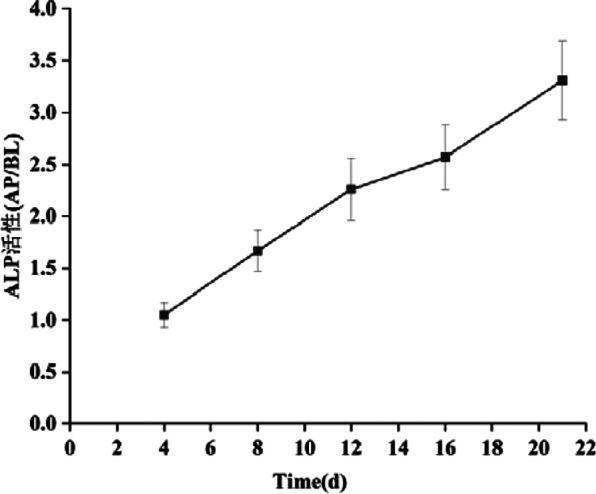
Changes of ALP activity ratio of Caco-2/HT29 co-culture cell model with culture time (‾x ± SD, n = 3).

The activity ratio of ALP serves as an indicator to evaluate the extent of cell differentiation towards an enterocyte phenotype (Calhau et al., 2002b). It is recognized as a marker of enterocyte differentiation that is independent of specific cell functions. Figure 14 illustrates the ALP activity ratio as it correlates with culture time in the Caco-2/HT29 cell model. ALP is predominantly secreted by cells of the brush border, and its activity increases as the cells further differentiate, particularly in the brush border region.

As depicted in the Figure 14, by day 4 of cell culture, the ALP activity ratio for both cell models had already exceeded 1. This suggests that the cell models had begun to exhibit preliminary polarization characteristics. By day 16, the ALP activity ratio in Caco-2 cells had risen above 3, indicating that the brush border of these cells was fully differentiated. By the 21st day, the viability of Caco-2 cells had gradually stabilized, and the ALP activity ratio for the Caco-2/HT29 co-cultured cells also exceeded 3. Consequently, it can be inferred that the distribution of ALP in both cell models is highly asymmetric and exhibits polar properties.

### 5.3 Mucus staining

**FIGURE 15:**
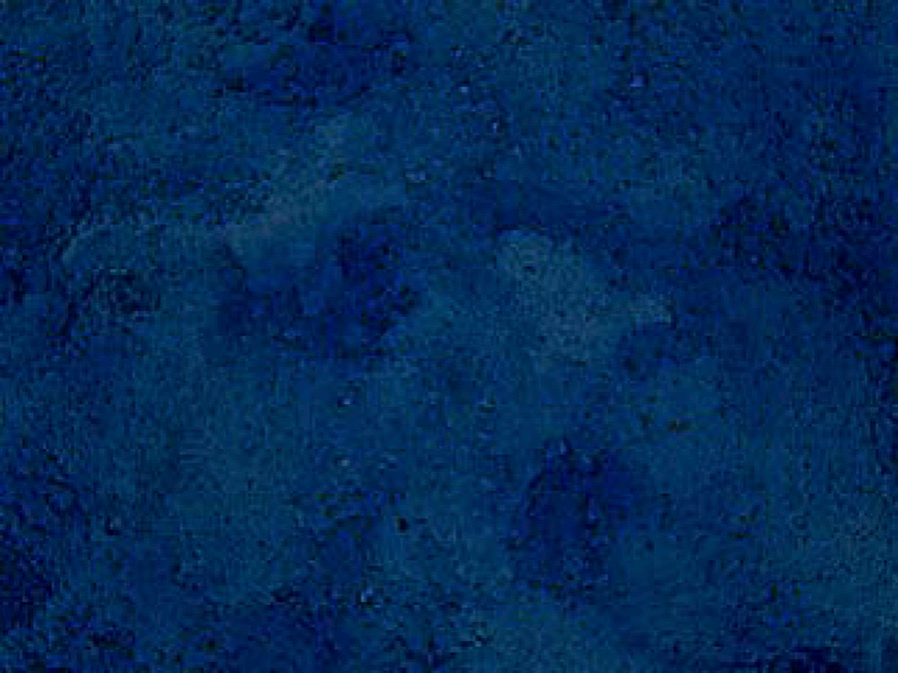
Alcian blue staining of Caco-2/HT29 co-cultured cells.

From Figure 15, it is confirmed that HT29 cells successfully produced mucus in co-culture with the Caco-2 cell model. The HT29-containing co-culture model can be utilized not only to study the penetration of active substances but also to more vividly demonstrate the impact of the mucus layer on uptake, as opposed to the traditional Caco-2 monolayer.

### 5.4 CCK8

**FIGURE 16:**
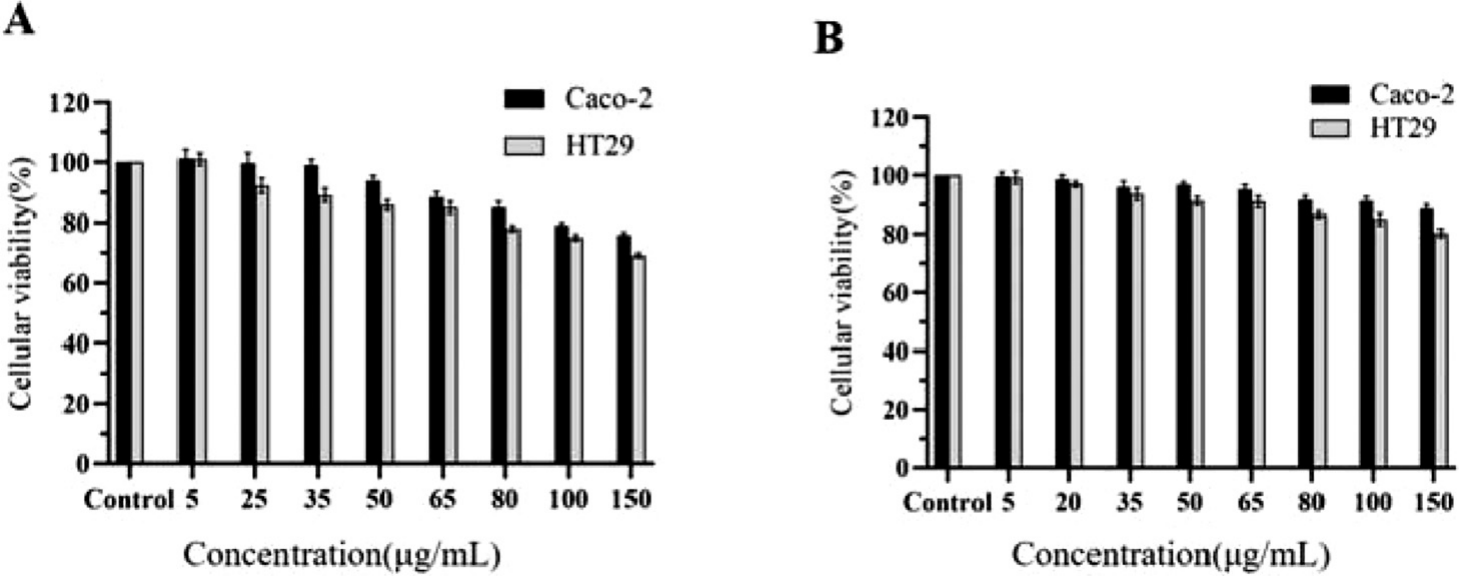
Cell viability of Caco-2 and HT29 cells treated with CA **(A)** and PUR **(B)** for 24 h, respectively (‾x ± SD, n = 6).

Compared to the control group treated with PBS, the activities of both cell types were influenced to varying extents by increasing drug concentrations. The CCK-8 assay results (Figure 16) indicated that the relative cell viability was maintained above 90% for CA at concentrations up to 25 μg/mL and for Puerarin at concentrations up to 65 μg/mL. Consequently, these concentration ranges are deemed suitable for further cellular transport experiments.

### 5.5 Transport of PUR

The *P*_*app*_ is a measure used to assess the extent of drug absorption. A *P*_*app*_ value exceeding 10^−5^cm/s typically indicates good drug absorption. However, the *P*_*app*_ values obtained in this experiment were all below 10^−5^cm/s, suggesting that Pur has poor absorption in the small intestine. Furthermore, the transport of the drug in the Caco-2/HT29 co-culture cell model was characterized by an efflux ratio (ER) greater than 2, which implies the presence of a transporter protein-mediated efflux mechanism in the transmembrane transport of Puerarin.

**FIGURE 17:**
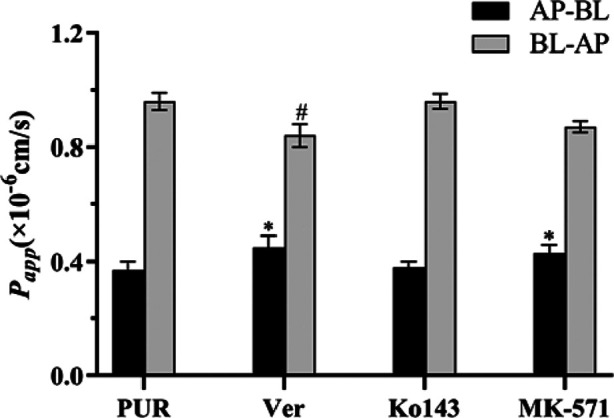
The effect of efflux transporter inhibitors on the transport of PUR, compared with the PUR group (AP-BL), *P < 0.05; compared with the PUR group (BL-AP),^#^ *P* < 0.05 (x ± SD, n = 3).

As demonstrated as Figure 17, the *P*_*app*_ values for the bidirectional transport of Pur following the application of three efflux inhibitors are presented. The total transport rate of Puerarin was significantly enhanced upon the addition of the P-gp inhibitor, verapamil, and the MRP inhibitor, MK-571 (*P* < 0.05). This enhancement suggests that both P-gp and MRP play a role in the transport mechanism of PUR.

According to the experimental outcomes, we observed that the transmembrane transport from the BL to the AP side was significantly reduced (*P* < 0.05) when PUR was combined with CA, as compared to PUR alone. Additionally, the ER value was also significantly lowered (*P* < 0.05). These results are detailed in Table 7. This finding implies that CA may diminish the secretory transport of PUR, thereby potentially enhancing its absorptive transport by inhibiting the exocytosis mediated by efflux transporters.

**TABLE 7:** Bidirectional transport results of PUR in the Caco-2/HT29 co-culture cell model with and without CA (‾x ± SD, n = 3).

	*P*_*app*_ (×10^−6^ cm/s)		ER
	AP-BL	BL-AP	
PUR	0.37	0.96	2.56
PUR + CA	0.47*	0.85*	1.82*

*P < 0.05, indicates a significant difference between the PUR group and the PUR + CA group.

## 6 Discussion and conclusion

With the progress of society and the enrichment and improvement of people’s material living standard, supplementing natural medicinal food has become a fashion, especially CR and PLR, as “medicine and food are homologous”, have attracted people’s attention and interest. CR is one of the commonly used TCM throughout history, widely used in clinical treatment. The main chemical constituents in CR are: cinnamaldehyde, CA, eugenol, gallic acid, coumarin, β-sitosterol, polymorphic glycosides, and several diterpenoids, which are consistent with our measured results (Wong et al., 2011; Maji et al., 2014; Xuan et al., 2023). Pharmacological studies have shown that CR have pharmacological effects such as regulating body temperature, analgesia, antibacterial, anti-inflammatory, anti-allergic, anti-viral, promoting vasodilation, diuretic, sedative, anti-anxiety, anti-tumor, lowering blood pressure, and treating diabetes. PLR is commonly used in the treatment of diabetes in TCM, “Shennong’s Classic of Materia Medica” recorded that: “PLR mainly treats diabetes, body heat, vomiting, all kinds of paralysis, raising “yin qi,” and relieving all kinds of poisons.” Modern pharmacological research reveals that PLR has shown positive effects in lowering blood sugar and blood lipids, resisting oxidative stress, protecting and nourishing the liver, and improving cardiovascular and cerebral vascular functions. PUR, the main active ingredient of PLR, can exert glucose-lowering effects through various pathways such as protection of pancreatic β-cells, improvement of insulin resistance, inhibition of α-glucosidase, anti-oxidative stress, anti-inflammation, and has demonstrated the advantages of synergistic effects of multi-targets in the treatment of T2DM and its complications (Wang et al., 2019). However, the chemical structural features of this class of substances result in poor lipid and water solubility of geraniol, which belongs to class IV compounds in the BCS classification system, affecting its bioactivity and pharmacological efficacy (Wang et al., 2020). PUR is poorly absorbed in the intestine, while the presence of P-gp and MRP transport protein-mediated exocytosis mechanisms in the transmembrane translocation of PUR is in accordance with the results of our cytological experiments (Cheng et al., 2020; Zhang, 2019). On the other hand, our results suggest that when PUR is combined with CA, CA may reduce the secretory transport of PUR, thereby enhancing its absorption transport by inhibiting the cytosolic effect mediated by the efflux transporters. Oral drugs are absorbed in the intestines and enter the blood circulation, and then distributed to various organs to exert therapeutic effects. The purpose of drug combination is to make more drugs enter the bloodstream and improve the bioavailability. Drugs entering the bloodstream must not only reach a certain concentration, but also be maintained for a long time to achieve therapeutic effects, pharmacokinetic experiments can be a good reflection of the difference between drug combination and single use. Pharmacokinetic experiments showed that the absorption of PUR was significantly increased when PLR was combined with CR. Cytological experiments showed that CA could enhance the uptake and transport of PUR by inhibiting P-gp and MRP efflux transporter-mediated efflux.

T2DM, which is mainly characterized by hyperglycemia and IR, is often accompanied by chronic complications in multiple organ systems, and its incidence is increasing year by year, making it a major challenge to human health (Zhou et al., 2023). Many macrogenomics and 16s rDNA microbial diversity-based studies have found that the host gut flora is significantly altered during pre-diabetes/metabolic syndrome and the development of T2DM (Le Chatelier et al., 2013; Karlsson et al., 2013; Qin et al., 2012). Firmicutes and Bacteroidota were the major phylum in all groups. Whereas Verrucomicrobiota was more predominant in the CR group, the Patescibacteria was more abundant in the PLR group. At the family level, the administration groups showed an increase in Ruminococcaceae and Lachnospiraceae, which are usually anaerobic bacteria belonging to the phylum Firmicutes and have been reported to be associated with the production of SCFAs, when compared with the model group (Vital et al., 2017; Weng et al., 2024; Zhang J. et al., 2019). In one study, Lachnospiraceae was shown to be a butyrate produce (Haas and Blanchard, 2017; Zhang J. D. et al., 2019). In contrast, Ruminococcaceae can ferment non-digestible carbohydrates (including polysaccharides, xylans, fibers, and resistant starch) to produce SCFAs, and is a key bacterium in the degradation of resistant starch (Ze et al., 2012).

SCFAs are mainly metabolites produced by bacterial fermentation of dietary fibers and are thought to play a key role in microbe-host cross-metabolism. SCFAs can influence organismal immunity as well as act as signaling molecules in cell growth processes in the colon (Wang et al., 2023; Li Y. et al., 2021; Karlsson et al., 2024). Several studies have shown that diets rich in SCFAs promote weight loss and improve glycemic control (Liu et al., 2018; Zhao et al., 2018), and some studies have reported supplementation with acetic, propionic, or butyric acids to improve energy metabolism in rodents (Fu et al., 2023; Lin et al., 2012). SCFAs can be directly activated to inhibit histone deacetylases (HDACs) and G Protein-Coupled Receptors (GPCRs) (Frolova et al., 2022), and inhibitors of HDACs have been widely used in cancer therapy, and SCFAs are considered as HDACs inhibitors. GPCRs, including GPR41, GPR43, and GPR109a, are sensitive receptors for SCFAs, and their activation is useful in metabolic disorders (Khan et al., 2024). Meanwhile, probiotics in the body (e.g., *Bacillus* spp. and *Lactobacillus* spp.) can produce SCFAs, such as acetic acid, propionic acid, and butyric acid, through fermentation of dietary fiber (Kimura et al., 2020). In addition, SCFAs can affect intestinal hormone secretion through the GPR41/GPR43-mediated signaling pathway (Hogh et al., 2020). GPR43, also known as free fatty acid receptor 2 (FFA2/FFAR2), is the main receptor for SCFAs, and propionate is the most important activator of GPR43 (Venegas et al., 2019). SCFAs can stimulate the secretion of GLP-1 and PYY by intestinal L cells through the activation of GPR43 (Kumar et al., 2020). Bjursell found that the SCFA/GPR43 pathway mediated an improvement in the levels of glucose-lipid metabolism in mice on a high-fat diet (Bjursell et al., 2011).

GLP-1, an enteroinsulinotropic hormone, is secreted by intestinal L cells and It has the ability to promote insulin secretion, inhibit glucagon secretion, delay gastric emptying, and reduce insulin-mediated glucose uptake to exert therapeutic effects on T2DM. The experimental results showed that rats in the model group had hyperglycemia, hyperlipidemia, IR, and decreased GLP-1 secretion. Interestingly, the intervention of CR and PLR restored the disordered state of STZ-induced T2DM rats, effectively reduced FBG, promoted GLP-1 secretion in the colon of T2DM rats, and improved glucose-lipid metabolism as well as IR.

In summary, CR-PLR can stimulate intestinal flora, increase the content of SCFAs, activate intestinal GPR43 protein, and promote the secretion of GLP-1 in intestinal L cells, which plays a therapeutic role in the treatment of type 2 diabetes. At the same time, pharmacokinetic studies suggest that CR can promote the absorption of puerarin and improve the efficacy, CA could enhance the uptake and transport of PUR by inhibiting P-gp and MRP efflux transporter-mediated efflux.

## Data Availability

The 16S rRNA sequencing data presented in the study are deposited in the NCBI SRA repository, accession number PRJNA1248667.

## Glossary

AUC: Area Under the Curve
T2DM: Type 2 Diabetes Mellitus
DM: Diabetes Mellitus
TCM: Traditional Chinese Medicine
STZ: Streptozotocin
UPLC-Q-TOF-MS: Ultra-Performance Liquid Chromatography coupled with Quadrupole Time of Flight Mass Spectrometry
LC-MS/MS: Liquid Chromatograph Mass Spectrometer/Mass Spectrometer
GC-MS: Gas chromatography Mass Spectrometry
HPLC: High Performance Liquid Chromatography
CR: Cinnamomi Ramulus
PLR: Puerariae Lobatae Radix
CR-PLR: Cinnamomi Ramulus- Puerariae Lobatae Radix
CA: Cinnamic acid
PUR: Puerarin
SCFAs: Short-chain fatty acids
PVDF: Polyvinylidene fluoride
TBS: Tris Buffered Saline
GPR43: G protein-coupled receptor 43
rpm: Revolutions per minute
GLP-1: Glucagon-like peptide-1
FBG: Fasting blood glucose
RSD: Relative standard deviation
FINS: Fasting serum lisulin
PBS: Phosphate buffer saline
SD: Standard deviation
S: Second
min: Minute
h: Hour
ELISA: Enzyme linked immunosorbent assay
IR: Insulin resistance
IL-1β: Interleukin- 1β
IL-6: Interleukin-6
TNF-a: Tumor necrosis factor-a
TC: Total cholesterol
TG: Triglyceride
HDL-C: High-density lipoprotein cholesterol
LPS: Lipopolysaccharide
PYY: Peptide tyrosine-tyrosine
GLP-1: Glucagon-like peptide-1
